# The Reporting Quality of Machine Learning Studies on Pediatric Diabetes Mellitus: Systematic Review

**DOI:** 10.2196/47430

**Published:** 2024-01-19

**Authors:** Zsombor Zrubka, Gábor Kertész, László Gulácsi, János Czere, Áron Hölgyesi, Hossein Motahari Nezhad, Amir Mosavi, Levente Kovács, Atul J Butte, Márta Péntek

**Affiliations:** 1 HECON Health Economics Research Center University Research and Innovation Center Óbuda University Budapest Hungary; 2 John von Neumann Faculty of Informatics Óbuda University Budapest Hungary; 3 Doctoral School of Innovation Management Óbuda University Budapest Hungary; 4 Doctoral School of Molecular Medicine Semmelweis University Budapest Hungary; 5 Doctoral School of Business and Management Corvinus University of Budapest Budapest Hungary; 6 Physiological Controls Research Center University Research and Innovation Center Óbuda University Budapest Hungary; 7 Bakar Computational Health Sciences Institute University of California San Francisco, CA United States

**Keywords:** diabetes mellitus, children, adolescent, pediatric, machine learning, Minimum Information About Clinical Artificial Intelligence Modelling, MI-CLAIM, reporting quality

## Abstract

**Background:**

Diabetes mellitus (DM) is a major health concern among children with the widespread adoption of advanced technologies. However, concerns are growing about the transparency, replicability, biasedness, and overall validity of artificial intelligence studies in medicine.

**Objective:**

We aimed to systematically review the reporting quality of machine learning (ML) studies of pediatric DM using the Minimum Information About Clinical Artificial Intelligence Modelling (MI-CLAIM) checklist, a general reporting guideline for medical artificial intelligence studies.

**Methods:**

We searched the PubMed and Web of Science databases from 2016 to 2020. Studies were included if the use of ML was reported in children with DM aged 2 to 18 years, including studies on complications, screening studies, and in silico samples. In studies following the ML workflow of training, validation, and testing of results, reporting quality was assessed via MI-CLAIM by consensus judgments of independent reviewer pairs. Positive answers to the 17 binary items regarding sufficient reporting were qualitatively summarized and counted as a proxy measure of reporting quality. The synthesis of results included testing the association of reporting quality with publication and data type, participants (human or in silico), research goals, level of code sharing, and the scientific field of publication (medical or engineering), as well as with expert judgments of clinical impact and reproducibility.

**Results:**

After screening 1043 records, 28 studies were included. The sample size of the training cohort ranged from 5 to 561. Six studies featured only in silico patients. The reporting quality was low, with great variation among the 21 studies assessed using MI-CLAIM. The number of items with sufficient reporting ranged from 4 to 12 (mean 7.43, SD 2.62). The items on research questions and data characterization were reported adequately most often, whereas items on patient characteristics and model examination were reported adequately least often. The representativeness of the training and test cohorts to real-world settings and the adequacy of model performance evaluation were the most difficult to judge. Reporting quality improved over time (*r*=0.50; *P*=.02); it was higher than average in prognostic biomarker and risk factor studies (*P*=.04) and lower in noninvasive hypoglycemia detection studies (*P*=.006), higher in studies published in medical versus engineering journals (*P*=.004), and higher in studies sharing any code of the ML pipeline versus not sharing (*P*=.003). The association between expert judgments and MI-CLAIM ratings was not significant.

**Conclusions:**

The reporting quality of ML studies in the pediatric population with DM was generally low. Important details for clinicians, such as patient characteristics; comparison with the state-of-the-art solution; and model examination for valid, unbiased, and robust results, were often the weak points of reporting. To assess their clinical utility, the reporting standards of ML studies must evolve, and algorithms for this challenging population must become more transparent and replicable.

## Introduction

### Background

In recent years, the regulatory authorization of medical devices and digital health technologies based on big data and machine learning (ML) has accelerated [1]. ML solutions have the potential to transform clinical practice by automating diagnosis, enhancing clinical decision-making, improving patient monitoring, and personalizing treatment [2]. Diabetes care has been among the first clinical areas to adapt ML technologies [1,2].

### Pediatric Diabetes Mellitus

Worldwide, pediatric diabetes mellitus (DM) is one of the most common chronic conditions among children, with growing incidence and increasingly complex presentation [3-6]. Type 1 DM (T1DM) is characterized by the lack of insulin secretion mainly due to autoimmune etiology, whereas type 2 DM (T2DM) is characterized by insulin resistance and metabolic syndrome associated with obesity [5]. Approximately 20% of children have both autoimmunity and insulin resistance [5]. A less common form, maturity-onset DM of the young, is attributed to a monogenic hereditary background [7,8]. Pediatric DM represents a difficult-to-treat population. Glucose targets frequently remain unmet in children and adolescents [9], and chronic complications, such as kidney disease, retinopathy, neuropathy, or hypertension, affect a significant proportion of patients by reaching young adulthood [10,11]. The life expectancy and quality of life of patients with pediatric DM may be reduced to a varying degree [5].

### ML in Pediatric DM

Within DM, pediatric DM has been leading the way to adopt digital technologies and intelligent devices [1,12]. Continuous glucose monitoring and automated insulin delivery systems are becoming an essential part of the management of children and adolescents with DM, with superior outcomes compared with alternative treatments [9,13]. Regulated smartphone apps for insulin dosing have become available for pediatric patients [14]. However, none of the currently available algorithms are optimal, and despite the use of advanced technology, many pediatric patients live under suboptimal glycemic control and are at risk of potentially serious long-term consequences [9,15]. In the quest for better disease characterization, prevention, and treatment of pediatric DM, ML has been increasingly applied from glucose sensors and artificial pancreas systems [16,17] to disease management apps (eg, mobile apps for food image–based carbohydrate counting or supporting self-management) [18,19] or risk prediction algorithms [20]. Although the comparison of novel algorithms has been challenging owing to methodological heterogeneity [9], artificial intelligence (AI) or ML algorithms have not been covered by DM technology guidelines [13,21,22]. To meet the needs of this challenging population, it is of utmost importance that algorithms are transparent and that their clinical value can be assessed.

### Reporting Quality of ML Studies in Medicine

Although expectations about the potential of technology to improve disease outcomes of pediatric DM have been rising [23,24], there has been growing concern about the transparency, replicability, biasedness, and overall validity of research in the field of AI and ML [25-30]. Indeed, examples of flawed or unfair predictions by algorithms and consequent legislative changes have sparked debate about the explainability, interpretability, and understandability of “black box” systems in ethical [31], philosophical [32,33], legal [34], social [35,36], computer [37], or medical sciences [38,39]. Although the concepts themselves remain vaguely defined or conflated [32], users in general and health care professionals in particular have sought explainable and interpretable ML models instead of predictions made by “black box” systems [40,41]. Although some ML models are “transparent” and others are “opaque” by nature, several post hoc techniques have been developed to make results interpretable, that is, to help medical professionals understand how and why machine decisions were made [40,41].

The outputs of ML models are probably more dependent on the input data than on the algorithm [42]. Therefore, the assessment of the fairness and accuracy of clinical ML studies should involve a thorough understanding of the processes of data production throughout the entire life cycle, from collection to annotation and processing [43]. Although technical aspects of data provenance may surpass the needs of clinicians, detailed reporting of the sources and production of data are indispensable for transparent and reproducible ML research and development [43].

We argue that high reporting quality standards are a prerequisite for the assessment and ultimately the achievement of methodological excellence in biomedical research. Although the association between incomplete reporting and biased treatment effects has been shown in clinical trials [44], evidence supports the positive effect of using checklists on reporting quality [45,46]. Despite the growing number and widespread adoption of reporting guidelines by leading journals over the past decades, deficient reporting of medical research studies remains a major concern, producing considerable waste [47].

Recognizing the limited usefulness of poorly reported studies in clinical practice, a plethora of reporting guidelines have been proposed for ML studies in medicine. Checklists have been developed for different study types (eg, observational studies, randomized trials, and health economic evaluations) and clinical areas aimed at standardizing the mandatory elements to be included in the study reports. These checklists are increasingly being used by scientific journal editors in medicine as mandatory elements for the submission of a manuscript [48]. Although targeting different apps and audiences, most reporting guidelines aim to ensure that results are reproducible, transparent, and, where appropriate, provide sufficient detail for inclusion in future evidence syntheses [26,30,48,49]. Hence, guidelines may contribute to the adoption of technologies with potential to benefit patients in real-world clinical settings.

### Research Aims

Various apps and methods of ML in diabetes care have been systematically reviewed [50,51], including specific use cases such as the prediction of hypoglycemia [52] or complications [53,54], diagnosis [55,56], use in disease management [24,57], and smart devices [58]. However, to the best of our knowledge, the reporting quality of ML studies in pediatric DM has not been systematically reviewed.

By acknowledging the potential of ML methods in addressing the specific treatment challenges of pediatric DM, we aimed to systematically review the reporting quality of ML studies on pediatric DM using a structured reporting checklist. Specifically, we aimed to highlight areas with adequate or poor reporting quality and identify the indicators of reporting quality. Furthermore, we explored the association of reporting quality with expert judgments about the overall clinical usefulness of the reported results.

## Methods

### Database Search

We considered the updated PRISMA (Preferred Reporting Item for Systematic Reviews and Meta-Analyses) 2020 statement when reporting the results of our study [59] (Multimedia Appendix 1). We searched the PubMed and Web of Science databases for the 5-year period from January 1, 2016, to December 31, 2020, using search syntaxes that combined the terms ML, children, and DM. For ML, we constructed a comprehensive search filter using the Medical Subject Headings (MeSH) terms of ML and AI [60,61]. We extended the search phrase with a list of terms from the caret package [62]. Given the rapidly expanding list and specialized use of methods, terms were added based on expert judgment. For studies on children, we adapted the Cochrane child search filter [63] by removing terms related to infants who were outside the scope of our study. In addition to the MeSH terms, the DM filter also included hyperglycemia, hypoglycemia, ketoacidosis, and insulin resistance. The detailed syntaxes and dates of the search in the PubMed and Web of Science databases are provided in Multimedia Appendix 2 and Multimedia Appendix 3, respectively.

### Screening and Selection of Eligible Studies

Original research reports published from 2016 to 2020 were eligible if ML methods were applied to analyze patient data on a population of children aged 2 to 18 years with DM of any subtype. As the primary research goal concerned the reporting quality of the applied ML methods, outcomes and interventions were not specified among the eligibility criteria. We restricted our review to a 5-year window to keep track of recent advances and maintain a feasible range. We included studies if DM or its complications (eg, retinopathy) were the primary diagnosis or DM was a study subpopulation (eg, population screening studies). If the relevant age group was covered, patients aged up to 25 years were accepted. We also included studies involving broader age groups if the results were reported separately for children. Both in vivo and in silico pediatric patients were allowed. No language restrictions were applied.

All records were independently screened by 12 pairs of reviewers formed by 6 authors. An extensive list of ML methods was provided to aid in record screening. Differences were resolved by consensus. Records were excluded if ineligibility could be clearly stated and retained for full-text screening in case of uncertainty or insufficient information.

The full-text reports were independently screened by 12 pairs of reviewers. All eligibility criteria were recorded and had to be reconciled in case of disagreement. Eligibility for the ML criterion was as follows: (1) either a typical ML method was specified (eg, bagging, boosting, bootstrap aggregated models, decision tree, deep belief network, denoising autoencoder, ensemble methods, genetic programming, learning, long short-term memory, model tree, neural network, neuro-fuzzy, random forest, random tree, and support vector) or (2) the data analysis algorithm involved the ML workflow of training, validation, and testing of results using any algorithm including traditional regression methods. In case of uncertainty, a third reviewer (AM) with technical expertise in ML methods made the decision. The third reviewer was omitted only if reviewers mutually agreed on the presence of criterion 1 or the absence of both criterion 1 and 2. Interrater agreement of reviewers was monitored during the screening of records and selection full-text reports via absolute agreement and Cohen κ.

### Assessment of Reporting Quality

#### Minimum Information About Clinical AI Modelling Checklist

##### Overview

Given the potentially diverse application of ML in pediatric DM, we applied the Minimum Information About Clinical Artificial Intelligence Modelling (MI-CLAIM) checklist, a general-purpose reporting guideline for medical AI studies available from the EQUATOR (Enhancing the Quality and Transparency of Health Research) network [64]. MI-CLAIM has been developed to enable the assessment of clinical impact (including fairness and bias) and the replication of the technical design process of clinical ML studies. It comprises 17 binary “yes” or “no” items organized into 6 domains: study design (part 1, consisting of 5 items), data and model optimization (parts 2 and 3, consisting of 5 items), model performance (part 4, consisting of 3 items), model examination (part 5, consisting of 5 items), and reproducibility (part 6, consisting of 1 item). Two categorical items ask about the type of data and reproducibility of the entire model pipeline. The data types can be categorized as structured (ie, that can be defined and understood by researchers) or unstructured (ie, the lack of explicitly definable raw features, such as images, natural language, or time series). The reproducibility of the entire model-building pipeline is described by 4 levels: tier 1 (ie, complete sharing of the code), tier 2 (ie, allowing a third party to evaluate the code for accuracy and fairness and share the results of this evaluation), tier 3 (ie, release of a virtual machine for running the code on new data without sharing its details), and tier 4 (no sharing). In this paper, we will refer to MI-CLAIM items by denoting the domain and item number within the domain (eg, item 6.1 denotes reproducibility). To aid the reporting and review process, the MI-CLAIM checklist requires the recording of the page numbers of a paper where relevant information was found concerning the checklist items. To address missing information or inadequate reporting, notes must be taken for each item [64].

##### Assessment of Reporting Quality Using MI-CLAIM

In this study, MI-CLAIM was applied as follows. Six reviewers were organized into pairs involving a medical expert (ÁH, LG, MP, and ZZ) and an expert in computer science (GK, HMN, and JC), who independently evaluated eligible studies along all items of MI-CLAIM. MI-CLAIM was elaborated in group training sessions before commencing the assessment. For the 17 binary items, response options were “yes” or “no” and “unsure” for cases when information was provided by the authors, but the reviewers could not come to a firm conclusion about whether the reporting was sufficiently clear or detailed. The final ratings relied on the expert judgment and consensus of the involved reviewer pairs. For unanimous “yes” answers, supporting information from the papers was extracted, summarized, and provided in supplementary tables, but no comments were made about missing items or inadequate information. Only those studies were eligible for the evaluation via MI-CLAIM, which followed the typical ML workflow: data-driven model training and validation followed by model testing on a designated data set [64]. Studies in which ML algorithms were used in alternative workflows were not evaluated using MI-CLAIM.

We added two additional items to reflect the overall purpose of MI-CLAIM: (1) “Did the paper enable the direct assessment of clinical impact, including fairness and bias?” (clinical impact) and (2) “Can the technical design process of the paper be rapidly replicated?” (replicability). For all studies, item 1 was rated by a senior medical expert (GL) and item 2 was rated by 2 experts who were well versed in computer science and medical data analysis (GK and ZZ). Responses were captured on a 5-level Likert scale (strongly agree; agree; neither agree nor disagree; disagree; strongly disagree).

### Extraction of Additional Data Items

In addition to the assessment of reporting quality, the first author, publication year, Scimago subject category of the publication (engineering and medicine) [65], title, main goal, applied ML method, input data, characteristics of the pediatric training and testing samples, and key findings of the included papers were extracted by a single reviewer (ZZ). Quantitative characteristics were extracted (eg, sample size), and inductive coding was applied to group studies into categories according to their goals, features of the training and test samples, and input data.

### Evidence Synthesis

We provided qualitative summaries of the answers to the MI-CLAIM by study and item. For each study, we denoted the count of “yes,” “no,” and “unsure” answers as well as the categories for data type (item 2.5) and reproducibility (item 6.1) as the MI-CLAIM profile of a study. For quantitative analysis, reproducibility (item 6.1) was dichotomized as “any sharing” (tiers 1-3) and “no sharing” (tier 4). In addition, the count of “yes” ratings for each study was referred to as reporting quality.

We assessed the association between reporting quality and continuous, dichotomous, and polytomous study characteristics using Pearson correlation, 2-sample *t* test, and one-way ANOVA, respectively. The normality of the distribution of reporting quality was tested using the Shapiro-Wilk test. The association between study characteristics and reporting quality (ie, “yes” ratings) was assessed via cross-tabulation and the Fisher exact test for each MI-CLAIM item.

Furthermore, we evaluated the correlation between reporting quality and the overall expert assessment of clinical impact. The association between reproducibility (item 6.1) and overall expert assessment of replicability was tested using a 2-sample *t* test.

## Results

### Screening and Selection of Eligible Reports

**Figure 1:**
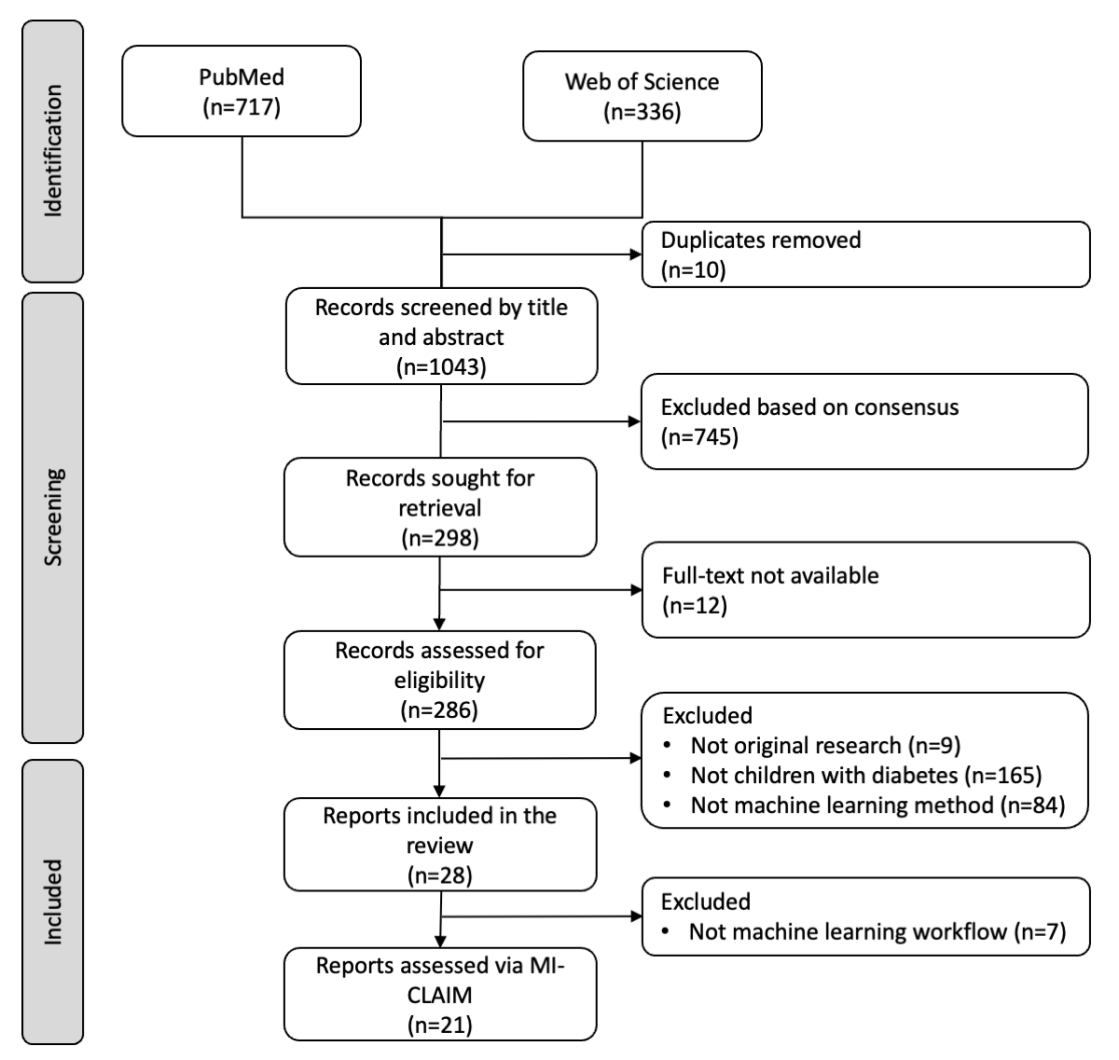
PRISMA (Preferred Reporting Items for Systematic Reviews and Meta-Analyses) flowchart of included studies. MI-CLAIM: Minimum Information About Clinical Artificial Intelligence Modelling.

The searches in PubMed and Web of Science databases yielded 717 and 336 records, respectively. After removing 9 duplicates, 1043 publication records were screened, 298 full-text reports were checked for eligibility, and 28 reports were eligible for our review. Research activity increased rapidly over time: 64% (18/28) of papers were published during the last 2 years, and 43% (12/28) were published in 2020 alone. (For web-based papers, the year of publication was subsequently updated in some cases). We assessed 21 reports using MI-CLAIM. Due to initial differences between reviewers, 20.4% (213/1043) of the records were reconciled. The absolute agreement and Cohen κ between reviewers’ initial judgments were 79.6% (range 63%-94%) and 0.47 (range 0.15 to 0.83), respectively. In the screening of full-text reports, 12 could not be retrieved due to lack of institutional access (Multimedia Appendix 4), 286 were assessed, 24.1% (69/286) were reconciled between reviewers, and in 13.6% (39/286) of the cases, a third reviewer was invited after the reviewers’ discussion to decide on the ML criterion. Altogether, the absolute agreement and κ of reviewers’ initial judgments regarding full-text selection were 67% (range 20%-85%) and 0.30 (range 0.06-0.30), respectively (Multimedia Appendix 5). Details of the search, screening, and inclusion are provided in the PRISMA (Preferred Reporting Items for Systematic Reviews and Meta-Analyses) flowchart (Figure 1).

### Characteristics of Included Studies

The characteristics of the 28 included studies are summarized in Table 1.

**Table 1:** Summary of included studies.

Study			Goal		ML^a^ method		Training sample		Test sample		Key finding
**ML methods applied with an ML workflow (reporting quality was assessed via MI-CLAIM^b^)**											
	Daskalaki et al [66], 2016	Develop an enhanced reinforcement learning model for personalized insulin delivery and glucose control: artificial pancreas system for pediatric T1DM^c^		Model-free actor-critic learning algorithm		In silico adolescents and children from a cohort of 28 patients from the UVA-Padova simulator; 2 outlier children were excluded: approximately 10 adolescents assumed and approximately 8 children. Four-day open-loop period followed by 5-d training.		5-d test period after training on the same in silico patient cohort.		In various parameter settings, time in range 77.8%-86.4% for adolescents and 74.8%-80.5% for children.	
	Ling et al [67], 2016	Detection of HG^d^ in patients with T1DM from the ECG^e^ signal		ELM-NN^f^ vs PSO-NN^g^, MR-FIS^h^, FIS^i^, and MR^j^		8 children (mean age 14.6, SD 1.5 y) with T1DM from a single center monitored overnight for nocturnal HG using CGM^k^ for 360-480 min		8 randomly selected patients with T1DM from the same center		On the basis of a linear combination of sensitivity and specificity (γ), ELM-NN was a superior classifier of HG vs other algorithms with a sensitivity of 0.78 and a specificity of 0.60	
	Miller et al [68], 2016	Determining subgroups of childhood-onset T1DM based on 25-y CVD^l^ risk		TSSA^m^		561 participants from the Pittsburgh Epidemiology of Diabetes Complication prospective cohort study; T1DM onset: age <17 y, between 1950 and 1980		Testing via k-fold cross-validation on the same patient cohort (k not reported)		Distinct subgroups for CVD risk exist within the childhood-onset T1DM cohort	
	Phyo et al [69], 2016	Detection of HG from ECG signal in pediatric T1DM		DBN^n^ vs BBNN^o^, WNN^p^, FFNN^q^, and MR		10 children with T1DM monitored 10h overnight for nocturnal HG using CGM from a single center (training and validation set)		5 randomly selected patients with T1DM from the same center		On the basis of a linear combination of sensitivity and specificity (γ), DBN was a superior classifier of HG vs other algorithms with a sensitivity of 0.80 and specificity of 0.50	
	Ling et al [70], 2017	Detect HG from ECG signal in patients with T1DM		Combinational MR-NLN^r^ with HPSOWM^s^ vs MR-NLN, NLN^t^, WNN, FFNN, and MR		From 15 T1DM children from a single center (12 with HG, mean age 14.6, SD 1.5 y), monitored 360-480 min overnight for nocturnal HG using CGM and ECG, 5/5 randomly selected for training and validation		5 randomly selected patients with T1DM from the same center		HG in children with T1DM can be detected from an ECG signal with a sensitivity of 0.79 and a specificity of 0.54	
	Siegel et al [21], 2017	Identify HG biomarkers from breath of children with T1DM.		LDA1^u^ with brute force feature selection by testing all possible combinations of predictors.		128 breath samples from 56 patients		Leave-one-out cross-validation using the training sample		HG can be predicted from the identified 7 volatile organic compounds with a sensitivity of 0.91 and a specificity of 0.84	
	Stawiski et al [71], 2018	Predict insulin resistance from sex, BMI, glucose, and lipid parameters in pediatric T1DM		NN^v^ (1000 random models) and MARSplines compared vs reference model (linear regression)		Patients with T1DM from a single center (N=252, mean age 14.95, SD 3.2 y); reference data: euglycemic hyperglycemic clamp		Patients with T1DM from the same center (N=63, mean age 15.15, SD 2.9 y)		NN and MARS^w^: better fit (*R*^2^) and accuracy (median error of prediction) vs reference model	
	Bois et al [72], 2019a	Compare the performance of ML algorithms in the prediction of HG on a 30-min horizon in pediatric T1DM		FFNN, LSTM^x^, ELM^y^, SVR^z^, GP-RBF^aa^, GP-DP^ab^		29-d observation of 10 in silico children using the UVA-Padova simulator, randomly split to 50% training and 25% validation data sets		25% randomly selected data sets from the same 10 in silico patients		GP^ac^ with dot-product kernel provided lowest RMSE^ad^ and best clinical accuracy on CG-EGA^ae^ with approximately 99% acceptable predictions for euglycemia	
	Bois et al [73], 2019b	Develop a model to improve the accuracy of long-term (120 min) glucose predictions in pediatric T1DM		A DCP^af^ model with parameter estimates using FFNN, GP with dot-product kernel, and ELM vs 2 alternative predictors: ACP^ag^ and AWA^ah^		28-d data from 10 in silico T1DM children from UVA-Padova simulator. 75% of data used for training in 4-fold cross-validation		25% of the data set from the same 10 in silico patients used for testing in 4-fold cross-validation		DCP with novel loss function had promising performance in long-term glucose predictions, with improved clinical acceptability (85.5% accurate predictions CG-EGA)	
	Khusial et al [74], 2019	Develop a plasma screening panel for NAFLD^ai^ in children using metabolomic data		Correlation-based feature selection, then SVM feature selection (information gain), then classification by LR^aj^, NB^ak^, and RF^al^		Sample size approximately 373; 2 of 3 of the total 559 patients aged 2-25 y, NAFLD: 222 (T2DM: 220), control 337 (T2DM: 328) from 3 studies: Emory University Pediatric Liver Biopsy Data repository, SweetBev Trial, and Yale Pediatric NAFLD Cohort		Sample size approximately 186 (1 of 3 out of the same 559 patients), selected randomly		RF predicted best NAFLD from metabolomic and clinical features with AUROC 0.94 (sensitivity of 0.73, specificity of 0.97)	
	Langner et al [75], 2019	Quantification of subcutaneous and visceral adipose tissue from MRI scans		2 CNN^am^ architectures: U-Net and V-Net		Patients with T2DM from the Tellus study (N=45, age 18-80 y)		Patients selected from Beta-JUDO study (N=10, age 10-18 y)		U-Net provided as accurate results in the test sample as literature reports of human operators and outperformed V-Net	
	Ngo et al [76], 2019	Detect nocturnal HG from EEG signals		BNN^an^		50% of data from 5 adolescents with T1DM (age 12-18 y) from a single-center overnight HG study (139 total episodes, 45 hypoglycemic)		50% of the same 5 adolescents (139 total episodes, 45 hypoglycemic)		Nocturnal HG could be detected from EEG signal with a sensitivity of 0.82 and specificity of 0.52	
	Stanfill et al [77], 2019	Develop a data preprocessing method (conditional classifier) to make classification algorithms applicable for omics data in matched case-control studies		Conditional LR, NB, SVM^ao^ with radial basis function kernel, SVM with linear kernel, RF, LDA2^ap^, and RPCLR^aq^ were compared		418 case-control pairs from The Environmental Determinants of Diabetes in the Young (TEDDY) study: genetic, lipidomic, metabolomic biomarkers of IA^ar^		5-fold cross-validation repeated 200 times on the same data set		Conditional SVM and NB outperformed LR in the classification of TEDDY data, with the potential to discover new biomarkers for IA	
	Amar et al [78], 2020	Compare a novel glucose prediction model vs alternative algorithms on real-life and in silico patients with T1DM		GCN1-3^as^ vs ARM^at^, RF, GBM^au^, FC^av^		In silico: 10 adolescents, 10 children (30-d training, 7-d validation) from UVA-Padova simulator; clinical: retrospective CGM data from 141 patients with T1DM from a single center (mean age 13.5, SD 5.2 y), mean (SD) CGM time: 64.4 (46.6) d		In silico: 7 d test on the same cohort; clinical: 4 d test on the same patients		Clinical accuracy 99.3% for 30-min and 95.8% for 60-min glucose predictions using CG-EGA, improved performance vs the standard ARM	
	Dave et al [79], 2020	Predict HG from glucose sensor data in pediatric T1DM		LR with LASSO^aw^ feature selection, and RF classifiers used on 26 extracted features from CGM data		Sample size approximately 78 (70% of data from 112 children) with T1DM (mean age 12.7, SD 4.8 y), 90 d follow-up using Dexcom G6 CGM device		Sample size approximately 34 (30% of data randomly selected from 112 children) in 10 replications		RF identified 30-60 min HG with a sensitivity of >0.91 and specificity of >0.90	
	Frohnert et al [80], 2020	Prediction of the development of IA and T1DM from genetic, immunologic, metabolomic, and proteomic biomarkers		ROFI-P3 integrative ML: combining optimal classifiers (LR, RF, KNN, LDA^2^, SVM, and NB) with iterative feature set selection for best predictive performance		67 children from the Diabetes Autoimmunity Study in the Young (DAISY) cohort, 22 with T1DM, 20 with persistent IA and 25 from control		The model was tested via 5-fold cross-validation on the training sample		Predictors for IA (ROC AUC^ax^ 0.91) and T1DM (ROC AUC 0.92) were identified and should be further validated	
	Garavelli et al [81], 2020	Identify circulating plasma microRNA with prognostic value for the progression of pediatric T1DM (stratification based on C-peptide loss)		FFNN for classification		150 children with T1DM: recent onset (n=88), 12 mo postdiagnosis (n=32), 24 mo postdiagnosis (n=30) from a single center		Two external validation cohorts: 18 children with recent onset T1DM and 26 children with recent onset T1DM from 2 separate clinical centers		Plasma microRNA can be used for the prognostic stratification of children with T1DM based on C-peptide loss	
	Li et al [82], 2020	GluNet: personalized 30- to 60-min glucose forecast in T1DM from CGM data, meal, insulin dose (GluNet)		DCNN^ay^ vs NN, LVR^az^ with exogenous input, ARM with exogenous input, SVR		10 in silico adolescents (UVA-Padova simulator); 90-d observation		The same 10 in silico adolescents, 90-d observation		DCNN improved RMSE, MARD^ba^, and time lag of glucose forecast vs alternative methods	
	Zhu et al [83], 2020	Develop and insulin bolus calculator planned for a smartphone app connected to CGM: artificial pancreas system for pediatric T1DM		DRL^bb^ vs standard insulin bolus calculator		10 in silico adolescents (UVA-Padova simulator), long-term general training, 180 d of personalized training		Same as the training cohort: after personalized training, 90 d testing with identical parameter settings for the test and control cohorts		DRL improved time in range (61.6%) vs the standard insulin bolus calculator (54.9%)	
	Zhu et al [84], 2020	Single-hormone (insulin) and dual-hormone (insulin+glucagon) dosing algorithm for closed-loop glucose control of T1DM		Dual-hormone DRL vs single hormone DRL vs state-of the art LGS^bc^ control strategy		10 in silico adolescents (UVA-Padova simulator), 1500 d generalized training followed by 30 d personalized training		Testing personalized models in the same cohort for 90 d (10 in silico adolescents)		Best time in range for dual-hormone DRL (78.8%) followed by single hormone DRL (65.9%) followed by LGS (55.5%)	
	Webb-Robertson et al [85], 2021	Predicting the development of IA and T1DM from metabolomic markers		Integrative ML: product of posterior probability and ROFI^bd^		236 children (118 matched pairs with or without islet autoimmunity or T1DM) from the TEDDY study		78 randomly selected children from the TEDDY study		42 predictor markers identified for T1DM, associated with 3 biological pathways	
**ML methods applied without an ML workflow (reporting quality was not assessed via MI-CLAIM)**											
	Alfonsi et al [18], 2020	Test the usability and impact on carbohydrate counting accuracy of an ML-based carbohydrate counting app (iSpy) in pediatric T1DM		CNN to identify food images		NR^be^		NR		Good acceptability, improved carbohydrate accuracy, and fewer counting errors and better HbA1c^bf^ vs controls	
	Adabimohazab et al [86], 2016	Explore the role of inflammation in the pathogenesis of insulin resistance among adolescents with obesity		RF used for classification		345 adolescents (mean age 17.5, SD 1.8 y), lean and overweight or obese		NR		No connection was found between low-grade inflammation and the development of insulin resistance	
	Brugha et al [87], 2018	Predict cystic fibrosis–related DM from CGM data		Hierarchical and k-means cluster analysis		142 children (age <17 y) with cystic fibrosis from a single center using CGM		NR		Greater median and IQR values for glucose fluctuation predict DM or prediabetes in children with cystic fibrosis	
	Cabrera et al [88], 2018	On the basis of immunoregulatory profiles, identify pediatric T1DM subtypes at clinical onset to predict postonset insulin secretion and responsiveness to abatacept therapy.		RF, hierarchical clustering		116 children with T1DM, within 100 d from diagnosis (62 control, 54 treated with abatacept)		NR		Innate inflammatory bias levels are associated with T1DM progression rate and responsiveness to abatacept.	
	Biassoni et al [89], 2020	Define gut microbial composition of new-onset pediatric T1DM		Supervised classification: RF, Elastic Net (L_1_L_2_); unsupervised analysis: WGCNA^bg^		New-onset T1DM pts (N=31, mean age 10.3, SD 4.1 y) vs sex-matched healthy controls (N=25, mean age 10.3, SD 4.1 y)		NR		New-onset T1DM has characteristic fecal microbial flora; not known if cause or consequence of autoimmunity	
	Nimri et al [90], 2020	Compare the performance of an AI-DSS^bh^ vs clinicians for insulin dosing in pediatric T1DM		DreaMed Advisor Pro, continuous glucose monitoring device with insulin pump, details of the ML method not provided		NR		NR		Time in range of AI-DSS was statistically noninferior when compared with physicians	
	Wolf et al [91], 2020	Economic evaluation of AI-based diabetic retinopathy screening vs standard care in pediatric DM from the patient perspective		Artificial intelligence–based diagnosis of diabetic retinopathy from digital fundus image. Methods not described in the paper		NR		NR		With >23% adherence to screening recommendations, artificial intelligence–based diabetic retinopathy screening is cost-saving for patients	

^a^ML: machine learning.

^b^MI-CLAIM: Minimum Information About Clinical Artificial Intelligence Modelling.

^c^T1DM: type 1 diabetes mellitus.

^d^HG: hypoglycemia.

^e^ECG: electrocardiogram.

^f^ELM-NN: extreme learning-based feed forward neural network.

^g^PSO-NN: particle swarm optimization based neural network.

^h^MR-FIS: multiple regression–based fuzzy inference system.

^i^FIS: fuzzy inference system.

^j^MR: multiple regression.

^k^CGM: continuous glucose monitor.

^l^CVD: cardiovascular disease.

^m^TSSA: tree-structured survival analysis.

^n^DBN: deep belief neural network.

^o^BBNN: block-based neural network.

^p^WNN: wavelet neural network.

^q^FFNN: feed forward neural network.

^r^MR-NLN: multiple regression–based neural logic network.

^s^HPSOWM: hybrid particle swarm optimization with wavelet mutation.

^t^NLN: neural logic network.

^u^LDA1: linear discriminant analysis.

^v^NN: neural network.

^w^MARS: multivariate adaptive regression splines.

^x^LSTM: long short-term memory.

^y^ELM: extreme learning machine.

^z^SVR: support vector regression.

^aa^RBF: radial basis function.

^ab^GP-DP: Gaussian process regression with dot-product kernel.

^ac^GP: Gaussian process regression.

^ad^RMSE: root mean square error.

^ae^CG-EGA: continuous glucose error grid analysis (Clarke error grid).

^af^DCP: derivatives combination predictor.

^ag^ACP: artificial neural network combination predictor.

^ah^AWA: adaptive weighted average fusion algorithm.

^ai^NAFLD: nonalcoholic fatty liver disease.

^aj^LR: logistic regression.

^ak^NB: naive Bayes.

^al^RF: random forest.

^am^CNN: convolutional neural network.

^an^BNN: Bayesian neural network.

^ao^SVM: support vector machine.

^ap^LDA2: latent Dirichlet allocation.

^aq^RPCLR: random penalized conditional logistic regression.

^ar^IA: islet autoimmunity.

^as^GCN: gradually connected neural network.

^at^ARM: autoregressive model.

^au^GBM: gradient boosting machine.

^av^FC: fully connected neural network.

^aw^LASSO: least absolute shrinkage and selection operator.

^ax^ROC AUC: receiver operating characteristic area under curve.

^ay^DCNN: dilated convolutional neural network.

^az^LVR: latent variable regression.

^ba^MARD: mean absolute relative difference.

^bb^DRL: deep reinforcement learning.

^bc^LGS: low glucose insulin suspension.

^bd^ROFI: repeated optimization for feature interpretation.

^be^NR: not reported.

^bf^HbA1c: hemoglobin A1c.

^bg^WGCNA: weighted correlation network analysis.

^bh^AI-DSS: AI-based decision support system.

Most studies (n=6) focused on the discovery of etiologic or prognostic biomarkers of T1DM [77,80,81,85,88,89], followed by studies aiming to predict hypoglycemia using noninvasive methods such as ECG or EEG signals, breath volatile organic compounds (n=5) [21,67,69,70,76], insulin bolus calculators for closed-loop glucose control (n=5) [66,82-84,90], accurate prediction of glucose levels or hypoglycemia from continuous glucose monitor (CGM) data (n=4) [72,73,78,79], etiologic or risk factors for insulin resistance or T2DM (n=4) [71,74,75,86], and other goals such as long-term cardiovascular risk stratification [68], accurate carbohydrate counting via a smartphone app [18], prediction of cystic fibrosis–related DM from CGM signal [87], and the economic evaluation of diabetic retinopathy screening via AI versus standard care [91].

### Reporting the Training and Test Samples

The characteristics of the training sample (including the validation data set) were not reported in 3 studies [18,90,91]. Of the 25 studies reporting details, in 18 (72%), the training sample involved human patients, in 6 (24%) only in silico patients [66,72,73,82-84], and there were both human and in silico patients in one study [78]. Of the 9 studies focusing on bolus calculation or glucose prediction from CGM data, only 2 (22%) had human patients in the training sample [78,79], 6 (67%) had only in silico patients [66,72,73,82-84], and the training sample was not characterized in one study [90]. The size of the training sample ranged from 561 [68] to 5 [76]. Of the 25 studies reporting details, only 10 (48%) had training samples with more than 100 patients [68,71,74,77,78,81,85-88], whereas 9 (36%) involved 10 or fewer patients [67,69,70,72,73,76,82-84]. Etiologic or prognostic studies for T1DM [77,80,81,85,88,89] and T2DM [71,74,75,86] featured the largest training samples ranging between 56 and 418 and 45 and 373, respectively. However, of the 14 studies focusing on insulin bolus calculators, or glucose or hypoglycemia prediction, only 4 (29%) [21,66,78,79] involved more than 10 patients in the training sample, with 141 being the largest sample size [78].

The test sample was characterized in 21 studies that involved a full ML workflow. Testing was performed on the same patients as the training in 12 (57%) studies, involving time-split in 8 studies using CGM data [66,72,73,76,78,82-84] and cross-validation in 4 studies [21,68,77,80]. In 5 (24%) studies, the test sample involved randomly selected patients from the same center as the training sample [67,69-71,79]; in 2 (10%) studies, patients from one or more external centers [75,81]; and in 2 (10%) studies randomly selected patients from multicenter studies [74,85]. The test sample included 10 or fewer patients in 10 (48%) studies [67,69,70,72,73,75,76,82-84], and only 4 (19%) studies featured test samples involving more than 100 patients [68,74,77,78]. The sample sizes of the external test samples ranged between 10 and 186 [74,75,81,85].

### ML Methods Used

In the 28 included studies, we identified a plethora of ML methods that sometimes overlapped with the techniques used for feature engineering, dimension reduction, or other steps of the analysis. Altogether, from the 87 proposed or comparator methods used, we identified 61 different techniques, with random forest (RF) mentioned in 8 studies [74,77-80,86,88,89], followed by feed forward neural network in 5 studies [69,70,72,73,81], logistic regression in 4 [74,77,79,80], and multiple regression (MR) [67,69,70] and naive Bayes in to 3-3 studies [77,80]. There were 48 methods mentioned in only one paper, and 2 papers did not specify the ML algorithm [90,91].

### Assessment of Reporting Quality Using the MI-CLAIM Profiles

**Figure 2:**
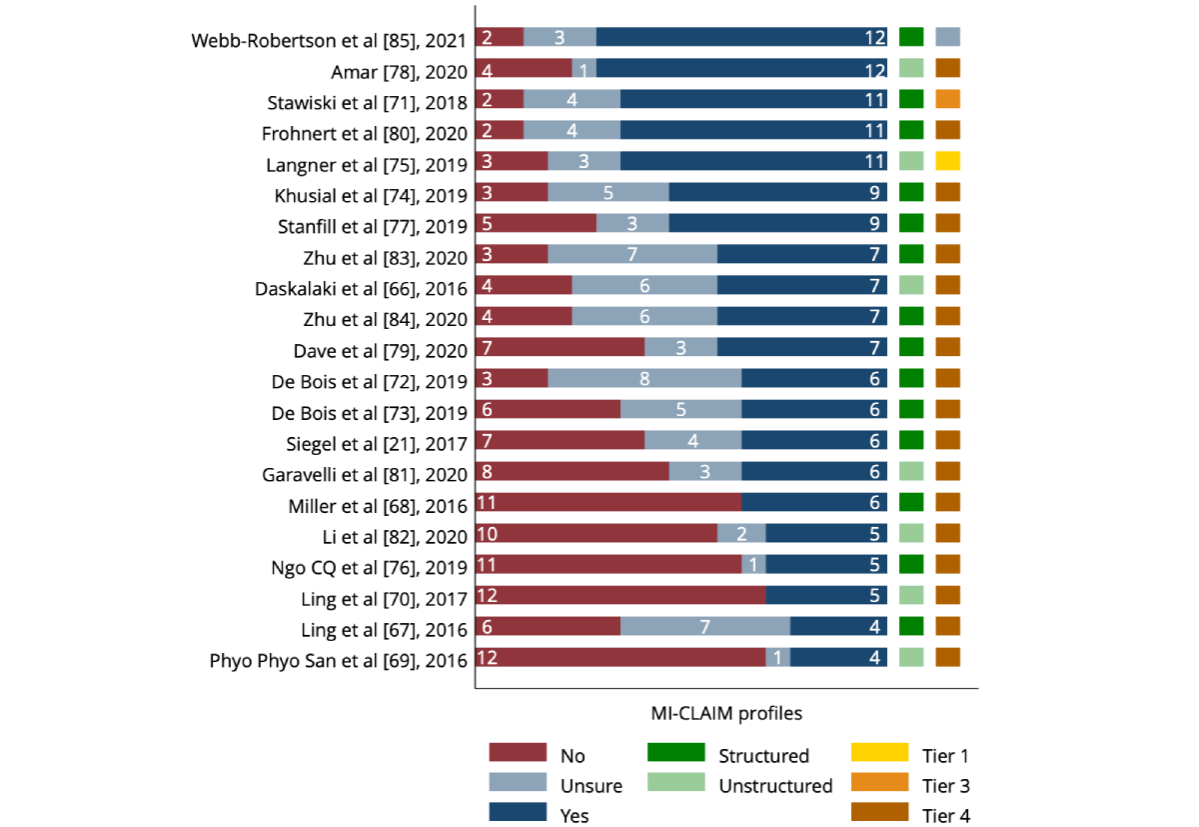
Minimum Information About Clinical Artificial Intelligence Modelling (MI-CLAIM) profiles by studies. MI-CLAIM profile: the count of “yes,” “no,” and “unsure” ratings for items 1.1 to 1.5, 2.1 to 2.4, 4.1 to 4.3, and 5.1 to 5.5; the classification of data type (item 2.5) as “structured” or “unstructured” and the classification of reproducibility (item 6.1) into tier 1 to 4 or “unsure.”.

The reporting quality via MI-CLAIM was assessed in 21 studies that followed the ML workflow. The characteristics of the assessed studies are summarized in Multimedia Appendix 6 [21,66-85]. The MI-CLAIM profiles for each study are shown in Figure 2 [21,66-85]. The assessment details of the included studies are provided in Multimedia Appendix 7 [21,66-85]. To support our “yes” ratings, the summaries of reported items for each study by the domains of MI-CLAIM are provided in Multimedia Appendices 8-11 [21,66-85].

For the 17 binary items, the number of “yes” ratings ranged between 4 and 12 (mean: 7.43), the “unsure” ratings ranged between 0 and 7 (mean 3.62), and the “no” ratings ranged between 2 and 12 (mean 6.95). One study provided a link to the applied unique ML model framework [85], without disclosing the specific code applied in the analysis. We rated reproducibility (item 6.1) for this study as “unsure” and dichotomized it as “any sharing.” The distribution of reporting quality was normal (Shapiro-Wilk test; *P*=.11). Reporting quality correlated positively with the year of publication, showing an improvement over time (*r*=0.50; *P*=.02). The reporting quality did not differ between studies using structured [21,67,68,70-74,76,77,79,80,83-85] or unstructured data [66,69,70,75,78,81,82] (*t*_19_=0.35; *P*=.73), or whether the input data were time series [66,69,70,72,73,78,79,82-84], omics [18,21,74,77,80,81,85], or other [68,71,75,76] (ANOVA *F*_2,18_=2.21; *P*=.14), in silico [66,72,73,82-84] or human subjects were involved [21,67-71,74-81,85] (*t*_19_=1.23; *P*=.24). However, it differed between studies with different research goals (ANOVA, *F*_5,15_=4.59; *P*=.01). Compared with the mean, prognostic biomarker studies in T1DM and risk factor studies of T2DM had significantly higher reporting quality by 2.02 (*P*=.04) and 2.85 (*P*=.01) “yes” ratings, respectively, whereas noninvasive hypoglycemia detection studies had fewer “yes” ratings by 2.69 (*P*=.006). Furthermore, higher reporting quality was observed in studies sharing any code of the ML pipeline [71,75,85] (*t*_19_=3.48; *P*=.003) and in studies published in medical journals [21,66,68,71,74,75,78-81,84,85] (*t*_19_=3.24; *P*=.004). Reporting quality correlated moderately with the overall expert assessment of clinical impact without significant association (*r*=0.40; *P*=.07).

### Assessment of Reporting Quality by the Items of MI-CLAIM

**Figure 3:**
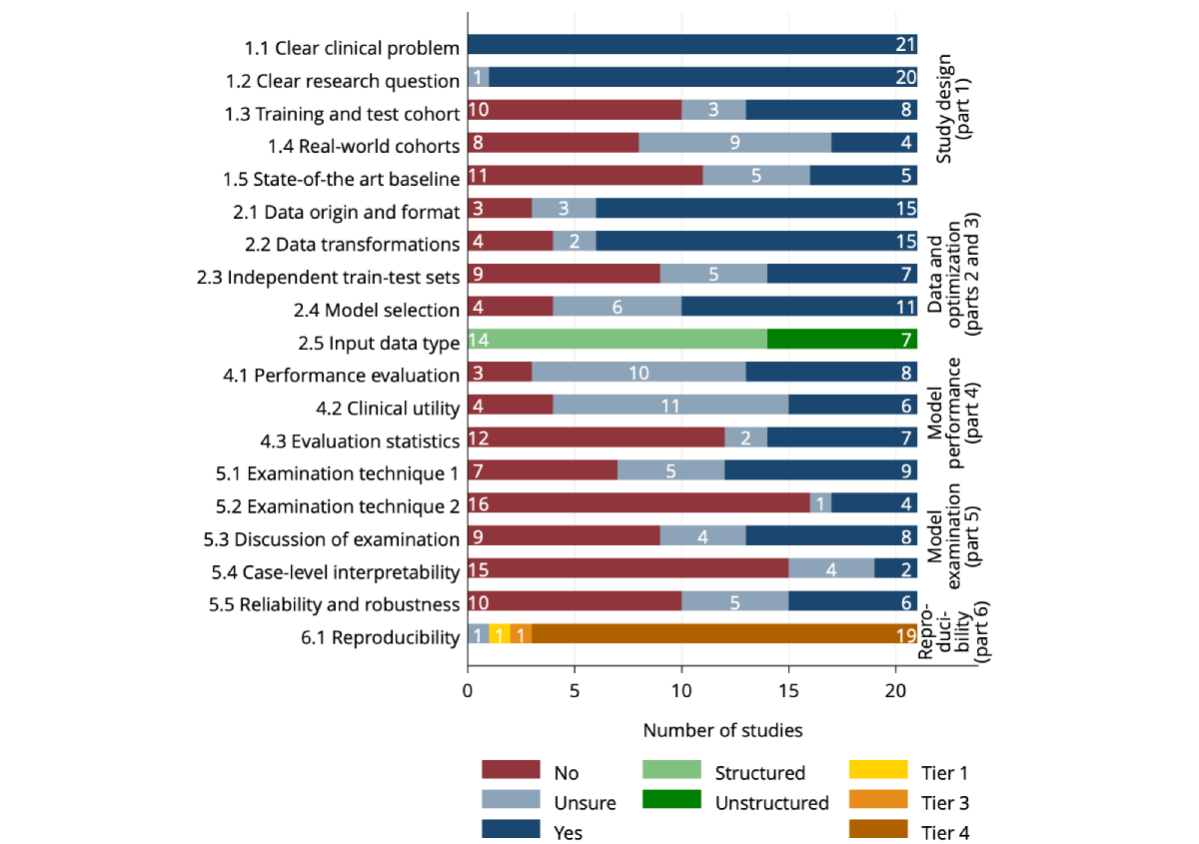
Assessment of reporting quality by Minimum Information About Clinical Artificial Intelligence Modelling (MI-CLAIM) items. The wording of MI-CLAIM items are as follows. Study design (part 1)—1.1: the clinical problem in which the model will be used is clearly detailed in the paper; 1.2: the research question is clearly stated; 1.3: the characteristics of the cohorts (training and test sets) are detailed in the text; 1.4: the cohorts (training and test sets) are shown to be representative of real-world clinical settings; 1.5: the state-of-the-art solution used as a baseline for comparison has been identified and detailed. Data and optimization (parts 2 and 3)—2.1: the origin of the data is described and the original format is detailed in the paper; 2.2: transformations of the data before they are applied to the proposed model are described; 2.3: the independence between training and test sets has been proven in the paper; 2.4: details on the models that were evaluated and the code developed to select the best model are provided; 2.5: is the input data type structured or unstructured? Model performance (part 4)—4.1: the primary metric selected to evaluate algorithm performance (eg, area under the curve and F score), including the justification for selection, has been clearly stated; 4.2: the primary metric selected to evaluate the clinical utility of the model (eg, positive predictive value and number needed to treat), including the justification for selection, has been clearly stated; 4.3: the performance comparison between the baseline and proposed model is presented with the appropriate statistical significance. Model examination (part 5)—5.1: examination technique 1a; 5.2: examination technique 2a; 5.3: a discussion of the relevance of the examination results with respect to model or algorithm performance is presented; 5.4: a discussion of the feasibility and significance of model interpretability at the case level if examination methods are uninterpretable is presented; 5.5: a discussion of the reliability and robustness of the model as the underlying data distribution shifts is included. Reproducibility (part 6)—choose the appropriate tier of transparency.

Figure 3 shows an assessment of reporting quality by the MI-CLAIM items [21,66-85].

#### Study Design (Part 1)

The clinical problem (item 1.1) was clearly defined in all studies, and the research question was clearly stated (item 1.2) in nearly all cases. However, in 1 (5%) study [79], our rating was uncertain about the clarity of the main research question. The characteristics of the training and test cohorts were clearly described (item 1.3) in 8 (38%) studies [68,71-74,78,81,85], our rating was “unsure” in 3 (14%) studies [75,83,84]. The cohorts were poorly characterized in all insulin bolus calculator and noninvasive hypoglycemia detection studies, receiving only “no” ratings (Fisher exact test; *P*=.03). Cohort characteristics were reported similarly in silico and in human studies. Our ratings were rather uncertain about whether the representativeness of the cohorts in real-world clinical settings was demonstrated (item 1.4). Only 4 (19%) studies received a “yes” rating [68,71,78,81], involving human sample sizes ranging between 140 and 561. Nine studies were rated as “unsure” (43%) [66,67,72,74,80,82-85], and in 8 (38%) studies, the representativeness of the sample in real-world situations was not demonstrated [21,69,70,73,75-77,79]. The state-of-the-art solution was not included as baseline (item 1.5) in 11 (52%) studies [21,66,68,71,72,75,76,79,81,82,85], and we were uncertain in 5 (24%) studies [67,69,73,74,78].

#### Data and Optimization (Parts 2 and 3)

Data origin and format (item 2.1) and data transformations (item 2.2) were described in detail in 15 (71%) studies [21,68,71-77,79,80,82-85] and 15 (71%) studies [66-68,71-75,77-79,81,82,84,85] with “unsure” rating in 3 (14%) [66,67,81,89] and 2 (10%) studies [80,83], respectively. The independence of the training and test samples was proven (item 2.3) in 7 (33%) studies [67,70,71,74,75,81,85], and we were “unsure” in 5 (24%) studies [72,73,82-84]. Reporting on this item differed according to the study aim (Fisher exact test; *P*=.04). The independence of the test and training samples was not demonstrated in studies focusing on insulin bolus calculation [66,82-84] or hypoglycemia detection from CGM data [72,73,78,79]. Item 2.4 requires that the details of the evaluated models and the code to select the best model are detailed. For this item, 11 (52%) studies received “yes” [21,71-73,76,78-80,83-85], and 6 studies received “unsure” (29%) rating [66,67,74,75,77,81]. The data were structured in 14 (67%) studies and unstructured in 7 (33%) studies. Our reviewers rated the 8 studies using glucose time-series data as structured in 5 (63%) and unstructured in 3 (38%) cases. This suggests that our reviewers’ notions varied regarding the direct interpretability of glucose time-series data (ie, as structured data were defined by the authors of MI-CLAIM) [64].

#### Model Performance (Part 4)

The primary metric for the evaluation of model performance was clearly stated and justified (item 4.1) in 8 (38%) studies [21,69-71,77,78,80,85], whereas our rating was frequently “unsure” (10/21, 48%) [66,67,72,74-76,79,81,83,84]. The reporting in this item differed between human and in silico studies (Fisher exact test; *P*=.046), with no in silico studies rated as “yes.” The selection and justification of the primary metric of clinical utility (item 4.2) received 6 (29%) “yes” [69,75,76,78,80,85], and 11 (52%) “unsure” ratings [21,66,67,71-74,77,79,83,84]. The performance comparisons were presented with appropriate statistical significance in only 7 (33%) studies [21,77,78,80,82-84], whereas the rating was “no” in over half of the studies (12/21, 57%) [66,68-70,72-76,79,81,85].

#### Model Examination (Part 5)

Model examination techniques help to validate that model accuracy is related to relevant inputs and explain how complex models work (eg, quantify the importance of predictor variables or characterize subjects with the best or poorest model performance) [64]. While one examination technique (item 5.1) was applied in 9 (43%) studies [66,71,74,75,77-80,85], a second examination technique (item 5.2) was applied in only 4 (19%) [75,77,80,85]. The use of at least one examination technique was more frequent among publications in medical journals (Fisher exact test; *P*=.02). Although model examination was not reported in any of the noninvasive hypoglycemia studies (Fisher exact test; *P*=.04), 2 examination techniques were reported only in prognostic biomarker studies in T1DM and risk factor studies of T2DM (Fisher exact test; *P*=.02) and mainly among studies using omics data (Fisher exact test; *P*=.03). MI-CLAIM suggests that examination results are more relevant for better-performing models and should be discussed in the context of model performance (item 5.3) [64], which was carried out in 8 (38%) studies [66,71,74,75,78-80,85], mainly published in medical publications (Fisher exact test; *P*=.005). Furthermore, if other examination techniques are infeasible, the results should be interpreted at the case level (item 5.4), which we found in only 2 (10%) studies [66,75]. Model reliability and robustness to shifts in data distribution was examined in 6 (29%) studies [66,74,75,78,79,83], and our rating was “unsure” in 5 (24%) cases [21,71,72,80,85]. Discussions on reliability and robustness were reported more often in medical publications (Fisher exact test; *P*=.02).

#### Reproducibility (Part 6)

All but 3 studies received “tier 4” rating, as the code of the ML workflow was not shared. One study shared the full code (“tier 1”), one provided a link to a downloadable calculator (“tier 3”) and one study received “unsure” rating. Our overall expert assessment of replicability did not differ between studies with “any sharing” and “no sharing” of the code of the model pipeline (*t*_19_=0.945; *P*=.36), suggesting that beyond the proposed tiers of MI-CLAIM, the reported technical details have influenced the replicability judgments.

## Discussion

### Principal Findings

This systematic review provides insights into reporting quality and, hence, the potential clinical impact of studies applying ML methods in pediatric DM populations. We applied the MI-CLAIM checklist to assess the reporting quality of 21 studies that followed the ML workflow of model training, validation, and testing. In these studies, reporting quality was generally low, with an improving trend over time. The MI-CLAIM items on research questions and data characterization were reported adequately most often, whereas the items on patient characteristics and model examination were reported adequately least often. The representativeness of the training and test cohorts to real-world settings and the adequacy of model performance evaluation were the most difficult to judge. On average, we found adequate reporting for less than half of the MI-CLAIM items, with considerable differences between studies with different research foci. Medical papers had higher reporting quality compared with articles published in engineering journals, mainly because of more elaborate reporting in the model examination domain. The number of MI-CLAIM items with a “yes” rating showed a moderate correlation with the overall assessment of the clinical impact by independent medical experts. We found no association between the reproducibility ratings on MI-CLAIM and independent experts’ assessments of the technical replicability of studies.

### Comparison With Prior Work

When writing this paper, MI-CLAIM was used in a single review, focusing on ML in dental and orofacial pain management, in which nearly all included papers were rated with “yes” in 13 or more out of the 15 assessed items [92]. Our study showed a less favorable picture with all studies having 12 or less “yes” ratings and two-thirds of studies having 7 or less “yes” ratings out of 17 items. Our findings corroborate the results of previous studies, raising concerns regarding the reporting quality of ML studies [25,27,29].

### Elaboration of Findings

Considering the globally increasing burden and serious consequences of pediatric DM [3] and the rapid growth of ML literature over the past years [48], we found few eligible studies, usually involving small patient populations. Of the 28 eligible studies, the training sample involved more than 100 patients in only 10 cases. In terms of research aims, applied methods, and data types, the studies were diverse.

According to our experience, compared with highly standardized medical papers, such as randomized clinical studies [93], systematic reviews [94], or economic evaluations [95], the reading and interpretation of the involved ML papers was challenging and time-consuming. The focus of MI-CLAIM on the clinical utility of ML modeling may explain the higher reporting quality of medical papers than those published in engineering journals. Still, the general reporting pattern reflected a “data-driven” mindset: after stating the clinical problem and research question, data-related items were reported most thoroughly. However, important details for clinicians, such as the detailed description of patient cohorts, the state-of-the-art clinical solution, and the clinical utility of the proposed models and model examination for valid, unbiased, and robust results were often the weak points of reporting.

We found an association between reporting quality and research goals. Studies with a strong clinical focus, such as those seeking prognostic biomarkers in T1DM and risk factors in T2DM, had higher reporting quality than the more technically oriented studies aimed at detecting hypoglycemia from CGM data or noninvasive methods or developing insulin bolus calculator algorithms. However, some of the reported differences were clearly attributable to the individual styles of the research teams. While reporting quality of the same teams, such as Webb-Robertson et al [77,80,85], Zhu et al [82-84], Bois et al [72,73], or Ling et al [67,69,70] fell in the same range, the number of “yes” ratings differed up to 6 items between different author teams in similar studies focusing on hypoglycemia prediction or the discovery of prognostic markers for T1DM.

Although MI-CLAIM was developed as a general reporting checklist, not as a measurement tool, our attempt to quantify reporting quality provided several learnings. We observed that the applicable ratings in some items depended on the underlying methods. For example, the independence of the training and test data could not be demonstrated in studies aimed at developing individualized glucose control algorithms. In contrast, the use of transparent models and a thorough examination of feature importance are hallmarks of prognostic marker studies, yielding naturally high scores in the model examination domain. Specific research questions, methods, or data types gave rise to specialized ML reporting guidelines for certain clinical fields or research designs [30,48]. Although MI-CLAIM provides a strong strategic framework for the evaluation of a broad range of ML applications, in our opinion, the evaluation of the validity and potential biases of ML studies in specific clinical use cases of pediatric DM, such as insulin bolus calculation or hypoglycemia detection from various physiological signals, would require more specific technical guidance, preferably from the medical profession, who provides the data and ultimately uses the results for clinical decision-making.

Some MI-CLAIM items received a high proportion of “unsure” ratings from our team. For example, item 1.4 suggests that the representativeness of training and test cohorts in real-world clinical settings should be demonstrated. Despite many studies reporting the parameter variability of T1DM simulators or the recruitment methods and characteristics of patient samples from diabetes clinics, we were unsure about what the established criteria were for representative cohorts of pediatric T1DM or T2DM. In addition, items 4.1 and 4.2 frequently received “unsure” ratings, which require that the primary evaluation metrics for model performance and clinical utility are clearly stated and justified. Although some authors “cherry picked” among multiple performance metrics to declare the superiority of the proposed model, in many cases, the assessment of adequate reporting was more challenging. For example, some studies applied the Clarke Error Grid Analysis [96] to evaluate the clinical accuracy of glucose predictions, reporting results in all 6 regions, but did not specify the primary region of interest (eg, potentially hazardous prediction errors). Other studies reported both sensitivity and specificity for hypoglycemia predictions without specifying a single measure. Furthermore, many studies have reported meaningful measures for the evaluation of model performance and clinical utility, without specifying their purpose. Examples include specificity and sensitivity, or the imaging study of Langner et al [75], which reported 3 metrics: the Dice score [97] and absolute and relative estimation error of adipose tissue volume. Although the metrics were adequate for both technical and clinical evaluation of results, owing to the lack of a single primary evaluation metric, the MI-CLAIM items could not be rated with unanimous “yes.” Furthermore, we found an overlap within the model examination domain, where the same piece of information satisfied multiple items, especially when the model examination involved performance testing in special patient populations.

Finally, we must note the diversity of methods and nomenclature used from data processing to feature selection, prediction, or evaluation of results. We found nearly 2 unique ML methods per paper, which makes the systematic search and evidence synthesis of ML challenging. While the term “machine learning” yielded 42,840 hits, our extended search term using specific methods provided 235,042 hits over our study’s search period in PubMed (Multimedia Appendices 2 and 11). While keeping track of the novel methods in ML is nearly impossible, the omission of specific terms carries the risk of incomplete search results for evidence synthesis. Furthermore, due to the specific methodological and reporting concerns about ML in medicine, we propose that the “machine learning study” label should be consistently used in the titles of medical studies, whose primary results arise from the typical data-driven ML workflow. This term has been used in the title or abstract of only 101 papers in PubMed in the same search period and could serve as unique identifier of clinical research using ML methods (Multimedia Appendix 12), benefiting future information retrieval and evidence synthesis similarly to the “randomized controlled trial” label.

### Limitations

A limitation of our study was that the literature search was closed in early 2021; therefore, recent publications potentially eligible for our review were not covered. In addition, given the plethora of ML methods, although the detailed list was included in the search syntax, some studies may have been missed during the search phase of our study. However, we believe that the key observations and conclusions of our review remain unaffected. In addition, we did not assess studies using MI-CLAIM, which reported ML results in pediatric DM without applying the ML workflow of model training, validation, and testing. Specific ML reporting guidelines exist [30,48] for clinical pilot studies [18], randomized controlled studies [90], and economic evaluations [91]. Despite the use of MI-CLAIM for a breadth of study types, we considered that MI-CLAIM was not applicable for a comparable assessment of those studies that omitted the steps of model validation and testing [86-89] with those that followed the full ML workflow.

Furthermore, despite the involvement of both medical experts and computer scientists in our research team, we observed high rates of disagreement between reviewers, and our consolidated ratings were “unsure” in over 20% of the assessed items. Although MI-CLAIM items were elaborated in group trainings, due to subjective judgments, some inconsistencies may have remained in our ratings. In particular, our judgments were unsure whether the representativeness of the training and test samples in real-world clinical settings was adequately demonstrated or if the selection and justification of primary model performance evaluation metrics were adequately justified. The specification of adequate sample characteristics and model performance evaluation criteria for clinical decision-making in pediatric DM and other disease areas remains an important area for future research.

### Conclusions

The reporting quality of ML studies in the pediatric population with DM was generally low. Important details for clinicians, such as the detailed description of patient cohorts, the state-of-the-art clinical solution, the clinical utility of the proposed models, and model examination for valid, unbiased, and robust results, were often the weak points of reporting. To allow the assessment of their clinical utility, it is of utmost importance that the reporting standards of ML studies evolve and algorithms for this challenging population become more transparent and replicable. MI-CLAIM provided a strong strategic framework for good reporting practices, which could be further supported by disease-specific technical guidance regarding what constitutes an adequate level of detail to inform clinical decision-making. Higher reporting quality standards may indirectly advance science and facilitate the uptake of technologies that have the potential to benefit children with DM.

## Appendix

Multimedia Appendix 1 PRISMA (Preferred Reporting Items for Systematic Reviews and Meta-Analyses) checklist.

Multimedia Appendix 2 Search syntax in PubMed.

Multimedia Appendix 3 Search syntax in Web of Science.

Multimedia Appendix 4 Excluded full-text papers.

Multimedia Appendix 5 Agreement between reviewers.

Multimedia Appendix 6 Characteristics of studies assessed via Minimum Information About Clinical Artificial Intelligence Modelling.

Multimedia Appendix 7 Assessment details of studies with Minimum Information About Clinical Artificial Intelligence Modelling.

Multimedia Appendix 8 Reported items in Minimum Information About Clinical Artificial Intelligence Modelling (part 1).

Multimedia Appendix 9 Reported items in Minimum Information About Clinical Artificial Intelligence Modelling (parts 2 and 3).

Multimedia Appendix 10 Reported items in Minimum Information About Clinical Artificial Intelligence Modelling (part 4).

Multimedia Appendix 11 Reported items in Minimum Information About Clinical Artificial Intelligence Modelling (part 5).

Multimedia Appendix 12 Additional searches in PubMed.

## Abbreviations

AI: artificial intelligence
CGM: continuous glucose monitor
DM: diabetes mellitus
MeSH: Medical Subject Headings
MI-CLAIM: Minimum Information About Clinical Artificial Intelligence Modelling
ML: machine learning
PRISMA: Preferred Reporting Items for Systematic Reviews and Meta-Analyses
RF: random forest
T1DM: type 1 diabetes mellitus
T2DM: type 2 diabetes mellitus
