# Supplementary material for: The Reporting Quality of Machine Learning Studies on Pediatric Diabetes Mellitus: Systematic Review
Source: J Med Internet Res. 2024 Jan 19;26:e47430. doi: 10.2196/47430 (PMC10837761; doi:10.2196/47430)
Supplement: Multimedia Appendix 2 [file jmir_v26i1e47430_app2.docx]

**Table S2 Search syntax in PubMed (Date: February 17^th^, 2021)**

| **#** | **Topic** | **Search term** | **Hits** |
| --- | --- | --- | --- |
| #1 | Machine Learning | „machine learning"[Title/Abstract] OR "neural network"[Title/Abstract] OR "neural networks"[Title/Abstract] OR "net reclassification"[Title/Abstract] OR "deep learning"[Title/Abstract] OR "long-short term memory"[Title/Abstract] OR "lstm"[Title/Abstract] OR "autoencoder"[Title/Abstract] OR "boltzmann machine"[Title/Abstract] OR "deep belief network"[Title/Abstract] OR "deep belief networks"[Title/Abstract] OR "artificial intelligence"[Title/Abstract] OR "support vector machine"[Title/Abstract] OR "decision tree"[Title/Abstract] OR "decision trees"[Title/Abstract] OR "computational intelligence"[Title/Abstract] OR "machine intelligence"[Title/Abstract] OR "artificial learning"[Title/Abstract] OR "connectionist model"[Title/Abstract] OR "connectionist models"[Title/Abstract] OR "random forest"[Title/Abstract] OR "computational reasoning"[Title/Abstract] OR "predictive algorithm"[Title/Abstract] OR "predictive algorithms"[Title/Abstract] OR "perceptron"[Title/Abstract] OR "perceptrons"[Title/Abstract] OR "computational intelligence"[Title/Abstract] OR "computer reasoning"[Title/Abstract] OR "ai artificial intelligence"[Title/Abstract] OR "computer vision systems"[Title/Abstract] OR "computer vision system"[Title/Abstract] OR "transfer learning"[Title/Abstract] OR "hierarchical learning"[Title/Abstract] OR "semi-supervised learning"[Title/Abstract] OR "expert systems"[Title/Abstract] OR "expert system"[Title/Abstract] OR "fuzzy logic"[Title/Abstract] OR "knowledgebases"[Title/Abstract] OR "knowledgebase"[Title/Abstract] OR "natural language processing"[Title/Abstract] OR ("knowledge bases"[Title/Abstract] AND ("computer"[Title/Abstract] OR "computers"[Title/Abstract])) OR ("knowledge base"[Title/Abstract] AND ("computer"[Title/Abstract] OR "computers"[Title/Abstract])) OR ("knowledge acquisition"[Title/Abstract] AND ("computer"[Title/Abstract] OR "computers"[Title/Abstract])) OR ("knowledge representation"[Title/Abstract] AND ("computer"[Title/Abstract] OR "computers"[Title/Abstract])) OR ("classification"[Title/Abstract] AND ("computer"[Title/Abstract] OR "computers"[Title/Abstract] OR "algorithm"[Title/Abstract] OR "algorithms"[Title/Abstract])) OR "artificial intelligence"[MeSH Terms] OR "machine learning"[MeSH Terms] OR "transfer, psychology"[MeSH Terms] OR "deep learning"[MeSH Terms] OR "supervised machine learning"[MeSH Terms] OR "computer heuristics"[MeSH Terms] OR "expert systems"[MeSH Terms] OR "expert systems"[MeSH Terms] OR "fuzzy logic"[MeSH Terms] OR "knowledge bases"[MeSH Terms] OR "natural language processing"[MeSH Terms] OR "neural networks, computer"[MeSH Terms] OR "ANFIS"[Title/Abstract] OR "neuro-fuzzy"[Title/Abstract] OR "neural fuzzy"[Title/Abstract] OR "neurofuzzy"[Title/Abstract] OR "fuzzy inference"[Title/Abstract] OR "nonlinear auto regressive"[Title/Abstract] OR "nonlinear autoregressive"[Title/Abstract] OR "artificial neural"[Title/Abstract] OR "autoregressive integrated moving average"[Title/Abstract] OR "ARIMA"[Title/Abstract] OR "back propagation neural"[Title/Abstract] OR "bagging"[Title/Abstract] OR "boosting machine"[Title/Abstract] OR "gradient boosting"[Title/Abstract] OR "catboost"[Title/Abstract] OR ("classification"[Title/Abstract] AND "decision trees"[Title/Abstract]) OR ("classification"[Title/Abstract] AND "decision tree"[Title/Abstract]) OR "continuous ranked probability score"[Title/Abstract] OR "convolutional neural"[Title/Abstract] OR "deep reinforcement learning"[Title/Abstract] OR "wavelet neural"[Title/Abstract] OR "fuzzy wavelet"[Title/Abstract] OR "recurrent wavelet"[Title/Abstract] OR "evolutionary computation"[Title/Abstract] OR "feed forward neural"[Title/Abstract] OR "boosting algorithm"[Title/Abstract] OR "k-means"[Title/Abstract] OR "LightGBM"[Title/Abstract] OR "long-short term memory"[Title/Abstract] OR "markov chain monte carlo"[Title/Abstract] OR "extreme learning machine"[Title/Abstract] OR "extreme learning machines"[Title/Abstract] OR "k-nearest"[Title/Abstract] OR "boltzmann machines"[Title/Abstract] OR "persistence model"[Title/Abstract] OR "persistence models"[Title/Abstract] OR "random matrix theory"[Title/Abstract] OR "auto encoder"[Title/Abstract] OR "autoencoder"[Title/Abstract] OR "support vector"[Title/Abstract] OR "XGBoost"[Title/Abstract] OR "computational intelligent"[Title/Abstract] OR "dimensionality reduction"[Title/Abstract] OR "radial basis function"[Title/Abstract] OR "TensorFlow"[Title/Abstract] OR "Matplotlib"[Title/Abstract] OR "Pandas"[Title/Abstract] OR "Keras"[Title/Abstract] OR "e1071"[Title/Abstract] OR "SciPy"[Title/Abstract] OR "Scikit-learn"[Title/Abstract] OR "Theano"[Title/Abstract] OR "TensorFlow"[Title/Abstract] OR "PyTorch"[Title/Abstract] OR "Orange3"[Title/Abstract] OR "NumPy"[Title/Abstract] OR " Scikit-Learn"[Title/Abstract] OR "Pandas"[Title/Abstract] OR "CARET"[Title/Abstract] OR "randomForest"[Title/Abstract] OR "Rpart"[Title/Abstract] OR "KernLab"[Title/Abstract] OR "nnet"[Title/Abstract] OR "dplyr"[Title/Abstract] OR "ggplot2"[Title/Abstract] OR "Wordcloud"[Title/Abstract] OR "tidyr"[Title/Abstract] OR "shiny"[Title/Abstract] OR "MICE Package"[Title/Abstract] OR "igraph"[Title/Abstract] OR "ROCR"[Title/Abstract] OR "DataExplorer"[Title/Abstract] OR "arules"[Title/Abstract] OR "mboost"[Title/Abstract] OR "MXNetR"[Title/Abstract] OR "darch"[Title/Abstract] OR "deepnet"[Title/Abstract] OR "deepr"[Title/Abstract] OR "KNIME"[Title/Abstract] OR "Xplenty"[Title/Abstract] OR "Weka"[Title/Abstract] OR "KNIME"[Title/Abstract] OR "Sisense"[Title/Abstract] OR "SSDT"[Title/Abstract] OR "Rapid Miner"[Title/Abstract] | 235,042 |
| #2 | Children | Child OR Child* OR Schoolchild* OR School age* OR Preschool* OR Kid OR kids OR Adolescent OR Adoles* OR Teen* OR Boy* OR Girl* OR Minors OR Minors* OR Puberty OR Pubert* OR Pubescen* OR Prepubescen* OR Pediatrics OR Paediatric* OR Paediatric* OR Peadiatric* OR Schools OR Kindergar* OR Primary school* OR Secondary school* OR Elementary school | 4,911,413 |

**Table S2 continued**

| #3 | Diabetes | "diabetes mellitus, type 1"[MeSH Terms] OR "diabetes mellitus, type 2"[MeSH Terms] OR "diabetes mellitus"[MeSH Terms] OR "diabetes type 1"[Title/Abstract] OR "diabetes type 2"[Title/Abstract] OR "diabetes mellitus"[Title/Abstract] OR ("diabete"[All Fields] OR "diabetes mellitus"[MeSH Terms] OR ("Diabetes"[All Fields] AND "mellitus"[All Fields]) OR "diabetes mellitus"[All Fields] OR "Diabetes"[All Fields] OR "diabetic"[All Fields] OR "diabetics"[All Fields] OR "diabets"[All Fields]) OR hypoglyc*[All Fields] OR hyperglic*[All Fields] OR ("ketosis"[MeSH Terms] OR "ketosis"[All Fields] OR "ketoacidosis"[All Fields]) OR "insulin resistance"[MeSH Terms] OR "insulin resistance"[Title/Abstract] | 997,914 |
| --- | --- | --- | --- |
| #4 | Date | ("2016/01/01"[Date - Publication] : "2020/12/31"[Date - Publication]) | 6,262,391 |
| #5 |  | #1 AND #2 AND #3 AND #4 | 717 |
