# Supplementary material for: The Reporting Quality of Machine Learning Studies on Pediatric Diabetes Mellitus: Systematic Review
Source: J Med Internet Res. 2024 Jan 19;26:e47430. doi: 10.2196/47430 (PMC10837761; doi:10.2196/47430)
Supplement: Multimedia Appendix 3 [file jmir_v26i1e47430_app3.docx]

**Table S3 Search syntax in Web of Science (Date: March 8^th^, 2021)**

| **#** | **Topic** | **Search term** | **Hits** |
| --- | --- | --- | --- |
| #1 | AI/ML | TI=("machine learning" OR " Scikit-Learn" OR ("classification" AND "decision tree") OR ("classification" AND "decision trees") OR ("classification" AND ("computer" OR "computers")) OR ("knowledge acquisition" AND ("computer" OR "computers")) OR ("knowledge base" AND ("computer" OR "computers")) OR ("knowledge bases" AND ("computer" OR "computers")) OR ("knowledge representation" AND ("computer" OR "computers")) OR "ai artificial intelligence" OR "algorithm" OR "algorithms" OR "ANFIS" OR "ARIMA" OR "artificial intelligence" OR "artificial learning" OR "artificial neural" OR "arules" OR "auto encoder" OR "autoencoder" OR "autoregressive integrated moving average" OR "back propagation neural" OR "bagging" OR "boltzmann machine" OR "boltzmann machines" OR "boosting algorithm" OR "boosting machine" OR "CARET" OR "catboost" OR "computational intelligence" OR "computational intelligent" OR "computational reasoning" OR "computer reasoning" OR "computer vision system" OR "computer vision systems" OR "connectionist model" OR "connectionist models" OR "continuous ranked probability score" OR "convolutional neural" OR "darch" OR "DataExplorer" OR "decision tree" OR "decision trees" OR "deep belief network" OR "deep belief networks" OR "deep learning" OR "deep reinforcement learning" OR "deepnet" OR "deepr" OR "dimensionality reduction" OR "dplyr" OR "e1071" OR "evolutionary computation" OR "expert system" OR "expert systems" OR "extreme learning machine" OR "extreme learning machines" OR "feed forward neural" OR "fuzzy inference" OR "fuzzy logic" OR "fuzzy wavelet" OR "ggplot2" OR "gradient boosting" OR "hierarchical learning" OR "igraph" OR "k-means" OR "k-nearest" OR "Keras" OR "KernLab" OR "KNIME" OR "knowledgebase" OR "knowledgebases" OR "LightGBM" OR "long-short term memory" OR "lstm" OR "machine intelligence" OR "markov chain monte carlo" OR "Matplotlib" OR "mboost" OR "MICE Package" OR "MXNetR" OR "natural language processing" OR "net reclassification" OR "neural fuzzy" OR "neural network" OR "neural networks" OR "neuro-fuzzy" OR "neurofuzzy" OR "nnet" OR "nonlinear auto regressive" OR "nonlinear autoregressive" OR "NumPy" OR "Orange3" OR "Pandas" OR "perceptron" OR "perceptrons" OR "persistence model" OR "persistence models" OR "predictive algorithm" OR "predictive algorithms" OR "PyTorch" OR "radial basis function" OR "random forest" OR "random matrix theory" OR "randomForest" OR "Rapid Miner" OR "recurrent wavelet" OR "ROCR" OR "Rpart" OR "Scikit-learn" OR "SciPy" OR "semi-supervised learning" OR "shiny" OR "Sisense" OR "SSDT" OR "support vector machine" OR "support vector" OR "TensorFlow" OR "Theano" OR "tidyr" OR "transfer learning" OR "wavelet neural" OR "Weka" OR "Wordcloud" OR "XGBoost" OR "Xplenty") OR AB=("machine learning" OR " Scikit-Learn" OR ("classification" AND "decision tree") OR ("classification" AND "decision trees") OR ("classification" AND ("computer" OR "computers")) OR ("knowledge acquisition" AND ("computer" OR "computers")) OR ("knowledge base" AND ("computer" OR "computers")) OR ("knowledge bases" AND ("computer" OR "computers")) OR ("knowledge representation" AND ("computer" OR "computers")) OR "ai artificial intelligence" OR "algorithm" OR "algorithms" OR "ANFIS" OR "ARIMA" OR "artificial intelligence" OR "artificial learning" OR "artificial neural" OR "arules" OR "auto encoder" OR "autoencoder" OR "autoregressive integrated moving average" OR "back propagation neural" OR "bagging" OR "boltzmann machine" OR "boltzmann machines" OR "boosting algorithm" OR "boosting machine" OR "CARET" OR "catboost" OR "computational intelligence" OR "computational intelligent" OR "computational reasoning" OR "computer reasoning" OR "computer vision system" OR "computer vision systems" OR "connectionist model" OR "connectionist models" OR "continuous ranked probability score" OR "convolutional neural" OR "darch" OR "DataExplorer" OR "decision tree" OR "decision trees" OR "deep belief network" OR "deep belief networks" OR "deep learning" OR "deep reinforcement learning" OR "deepnet" OR "deepr" OR "dimensionality reduction" OR "dplyr" OR "e1071" OR "evolutionary computation" OR "expert system" OR "expert systems" OR "extreme learning machine" OR "extreme learning machines" OR "feed forward neural" OR "fuzzy inference" OR "fuzzy logic" OR "fuzzy wavelet" OR "ggplot2" OR "gradient boosting" OR "hierarchical learning" OR "igraph" OR "k-means" OR "k-nearest" OR "Keras" OR "KernLab" OR "KNIME" OR "knowledgebase" OR "knowledgebases" OR "LightGBM" OR "long-short term memory" OR "lstm" OR "machine intelligence" OR "markov chain monte carlo" OR "Matplotlib" OR "mboost" OR "MICE Package" OR "MXNetR" OR "natural language processing" OR "net reclassification" OR "neural fuzzy" OR "neural network" OR "neural networks" OR "neuro-fuzzy" OR "neurofuzzy" OR "nnet" OR "nonlinear auto regressive" OR "nonlinear autoregressive" OR "NumPy" OR "Orange3" OR "Pandas" OR "perceptron" OR "perceptrons" OR "persistence model" OR "persistence models" OR "predictive algorithm" OR "predictive algorithms" OR "PyTorch" OR "radial basis function" OR "random forest" OR "random matrix theory" OR "randomForest" OR "Rapid Miner" OR "recurrent wavelet" OR "ROCR" OR "Rpart" OR "Scikit-learn" OR "SciPy" OR "semi-supervised learning" OR "shiny" OR "Sisense" OR "SSDT" OR "support vector machine" OR "support vector" OR "TensorFlow" OR "Theano" OR "tidyr" OR "transfer learning" OR "wavelet neural" OR "Weka" OR "Wordcloud" OR "XGBoost" OR "Xplenty") | 2,494,677 |
| #2 |  | TS=(Child OR Child* OR Schoolchild* OR School age* OR Preschool* OR Kid OR kids OR Adolescent OR Adoles* OR Teen* OR Boy* OR Girl* OR Minors OR Minors* OR Puberty OR Pubert* OR Pubescen* OR Prepubescen* OR Pediatrics OR Paediatric* OR Paediatric* OR Peadiatric* OR Schools OR Kindergar* OR Primary school* OR Secondary school* OR Elementary school) | 3,153,143 |
| #3 |  | TS=((diabetes AND mellitus) OR (diabetes AND type) 1 OR (diabetes AND type 2) OR "diabetic" OR "diabetics" OR "diabets" OR "hypoglyc" OR "hyperglic" OR "ketosis" OR "ketoacidosis" OR "insulin resistance”) | 710,860 |
| #4 |  | #1 AND #2 AND #3 | 636 |
| #5 |  | #1 AND #2 AND #3 Refined by: PUBLICATION YEARS: ( 2020 OR 2019 OR 2018 OR 2017 OR 2016 ) | 336 |
