# Supplementary material for: The Reporting Quality of Machine Learning Studies on Pediatric Diabetes Mellitus: Systematic Review
Source: J Med Internet Res. 2024 Jan 19;26:e47430. doi: 10.2196/47430 (PMC10837761; doi:10.2196/47430)
Supplement: Multimedia Appendix 4 [file jmir_v26i1e47430_app4.docx]

**Table S4 Excluded full-text papers**

| **Author, year** | **Journal** | **Title** | **Paper ID** | **Reason for exclusion** |
| --- | --- | --- | --- | --- |
| Althari A, 2020 | Am J Hum Genet | Unsupervised Clustering of Missense Variants in HNF1A Using Multidimensional Functional Data Aids Clinical Interpretation | 10.1016/j.ajhg.2020.08.016 | Not machine learning |
| Abraham MB, 2016 | Diabetes Technol Ther | Prevention of Insulin-Induced Hypoglycemia in Type 1 Diabetes with Predictive Low Glucose Management System | DOI: 10.1089/dia.2015.0364 | Not paediatric diabetes |
| Abraham MB, 2016 | Diabetes Technol Ther | Effectiveness of a Predictive Algorithm in the Prevention of Exercise-Induced Hypoglycemia in Type 1 Diabetes | DOI: 10.1089/dia.2016.0141 | Not machine learning |
| Adavi M, 2016 | Med J Islam Repub Iran | Artificial neural networks versus bivariate logistic regression in prediction diagnosis of patients with hypertension and diabetes | PMID: 27390682 | Not paediatric diabetes |
| Afarideh M, 2016 | Atherosclerosis | Complex association of serum alanine aminotransferase with the risk of future cardiovascular disease in type 2 diabetes | DOI: 10.1016/j.atherosclerosis.2016.09.009 | Not paediatric diabetes |
| Al-Taee MA, 2016 | Diabetes Technol Ther | Acceptability of Robot Assistant in Management of Type 1 Diabetes in Children | DOI: 10.1089/dia.2015.0428 | Not machine learning |
| Anderson AE, 2016 | J Biomed Inform | Electronic health record phenotyping improves detection and screening of type 2 diabetes in the general United States population: A cross-sectional, unselected, retrospective study | DOI: 10.1016/j.jbi.2015.12.006 | Not paediatric diabetes |
| Anjaneya LH, 2016 | 2016 IEEE International Conference on Recent Trends in Electronics, Information & Communication Technology | Multilayer machine learning algorithm to classify diabetic type on knee dataset | DOI: 10.1109/RTEICT.2016.7807889 | Not paediatric diabetes |
| Canamero L, 2016 | International Journal of Social Robotics | Making New "New AI" Friends: Designing a Social Robot for Diabetic Children from an Embodied AI Perspective | DOI: 10.1007/s12369-016-0364-9 | Not machine learning |
| Del Favero S, 2016 | Diabetes Care | Randomized Summer Camp Crossover Trial in 5-to 9-Year-Old Children: Outpatient Wearable Artificial Pancreas Is Feasible and Safe | DOI: 10.2337/dc15-2815 | Not machine learning |
| Elbarbary NS, 2016 | Diabetes-Metabolism Research and Reviews | Effectiveness of the low glucose suspend feature of insulin pump during fasting during Ramadan in type 1 diabetes mellitus | DOI: 10.1002/dmrr.2781 | Not machine learning |
| Endesfelder D, 2016 | Diabetologia | A novel approach for the analysis of longitudinal profiles reveals delayed progression to type 1 diabetes in a subgroup of multiple-islet-autoantibody-positive children | DOI: 10.1007/s00125-016-4050-0 | Not machine learning |
| Gawlik A, 2016 | J Clin Endocrinol Metab | Steroid Metabolomic Disease Signature of Nonsyndromic Childhood Obesity | DOI: 10.1210/jc.2016-1754 | Not paediatric diabetes |
| Hosseini SM, 2016 | Hum Brain Mapp | Altered Integration of Structural Covariance Networks in Young Children With Type 1 Diabetes | DOI: 10.1002/hbm.23293 | Not machine learning |
| Ivanović D, 2016 | J Med Syst | ANN Prediction of Metabolic Syndrome: a Complex Puzzle that will be Completed | DOI: 10.1007/s10916-016-0601-7 | Not paediatric diabetes |
| Khalyfa A, 2016 | Chest | Circulating microRNAs as Potential Biomarkers of Endothelial Dysfunction in Obese Children | DOI: 10.1378/chest.15-0799 | Not paediatric diabetes |
| Laffel L, 2016 | Diabetes Technology & Therapeutics | Improved Accuracy of Continuous Glucose Monitoring Systems in Pediatric Patients with Diabetes Mellitus: Results from Two Studies | DOI: 10.1089/dia.2015.0380 | Not machine learning |
| Ledford D, 2016 | Allergy Asthma Proc | Corticosteroid-related toxicity in patients with chronic idiopathic urticariachronic spontaneous urticaria | DOI: 10.2500/aap.2016.37.3999 | Not paediatric diabetes |
| Lee JB, 2016 | Industrial & Engineering Chemistry Research | Enhanced Model Predictive Control (eMPC) Strategy for Automated Glucose Control | DOI: 10.1021/acs.iecr.6b02718 | Not paediatric diabetes |
| Lu CL, 2016 | Environ Int | Inverse relationship between ambient temperature and admissions for diabetic ketoacidosis and hyperglycemic hyperosmolar state: A 14-year time-series analysis | DOI: 10.1016/j.envint.2016.06.032 | Not machine learning |
| Ly TT, 2016 | Diabetes Technology & Therapeutics | Automated Overnight Closed-Loop Control Using a Proportional-Integral-Derivative Algorithm with Insulin Feedback in Children and Adolescents with Type 1 Diabetes at Diabetes Camp | DOI: 10.1089/dia.2015.0431 | Not machine learning |

| **Author, year** | **Journal** | **Title** | **Paper ID** | **Reason for exclusion** |
| --- | --- | --- | --- | --- |
| Miotto R, 2016 | Sci Rep | Deep Patient: An Unsupervised Representation to Predict the Future of Patients from the Electronic Health Records | DOI: 10.1038/srep26094 | Not paediatric diabetes |
| Pan J, 2016 | Mol Vis | Serum molecular signature for proliferative diabetic retinopathy in Saudi patients with type 2 diabetes | PMID: 27307695 | Not paediatric diabetes |
| Patel NS, 2016 | Diabetes Technology & Therapeutics | Mitigating Reductions in Glucose During Exercise on Closed-Loop Insulin Delivery: The Ex-Snacks Study | DOI: 10.1089/dia.2016.0311 | Not paediatric diabetes |
| Pesenacker AM, 2016 | Diabetes | A Regulatory T-Cell Gene Signature Is a Specific and Sensitive Biomarker to Identify Children With New-Onset Type 1 Diabetes | DOI: 10.2337/db15-0572 | Not machine learning |
| Pitkänen N, 2016 | Diabetes Care | Role of Conventional Childhood Risk Factors Versus Genetic Risk in the Development of Type 2 Diabetes and Impaired Fasting Glucose in Adulthood: The Cardiovascular Risk in Young Finns Study | DOI: 10.2337/dc16-0167 | Not paediatric diabetes |
| Pitkanen N, 2016 | Diabetes Care | Role of Conventional Childhood Risk Factors Versus Genetic Risk in the Development of Type 2 Diabetes and Impaired Fasting Glucose in Adulthood: The Cardiovascular Risk in Young Finns Study | DOI: 10.2337/dc16-0167 | Not paediatric diabetes |
| Pyle L, 2016 | American Journal of Physiology-Endocrinology and Metabolism | Modeling changes in glucose and glycerol rates of appearance when true basal rates of appearance cannot be readily determined | DOI: 10.1152/ajpendo.00368.2015 | Not machine learning |
| Roychowdhury S, 2016 | 2016 38th Annual International Conference of the IEEE Engineering in Medicine and Biology Society | Classification of Large-Scale Fundus Image Data Sets: A Cloud-Computing Framework | DOI: 10.1109/EMBC.2016.7591423 | Not paediatric diabetes |
| Russell SJ, 2016 | Lancet Diabetes & Endocrinology | Day and night glycaemic control with a bionic pancreas versus conventional insulin pump therapy in preadolescent children with type 1 diabetes: a randomised crossover trial | DOI: 10.1016/s2213-8587(15)00489-1 | Not machine learning |
| Sharifi A, 2016 | Diabetes Technology & Therapeutics | Glycemia, Treatment Satisfaction, Cognition, and Sleep Quality in Adults and Adolescents with Type 1 Diabetes When Using a Closed-Loop System Overnight Versus Sensor-Augmented Pump with Low-Glucose Suspend Function: A Randomized Crossover Study | DOI: 10.1089/dia.2016.0288 | Not machine learning |
| Tauschmann M, 2016 | Diabetes Care | Home Use of Day-and-Night Hybrid Closed-Loop Insulin Delivery in Suboptimally Controlled Adolescents With Type 1 Diabetes: A 3-Week, Free-Living, Randomized Crossover Trial | DOI: 10.2337/dc16-1094 | Not machine learning |
| Tauschmann M, 2016 | Diabetes Care | Day-and-Night Hybrid Closed-Loop Insulin Delivery in Adolescents With Type 1 Diabetes: A Free-Living, Randomized Clinical Trial | DOI: 10.2337/dc15-2078 | Not machine learning |
| Tauschmann M, 2016 | Diabetes Care | Home Use of Day-and-Night Hybrid Closed-Loop Insulin Delivery in Suboptimally Controlled Adolescents With Type 1 Diabetes: A 3-Week, Free-Living, Randomized Crossover Trial | DOI: 10.2337/dc16-1094 | Not machine learning |
| Tauschmann M, 2016 | Diabetes Care | Day-and-Night Hybrid Closed-Loop Insulin Delivery in Adolescents With Type 1 Diabetes: A Free-Living, Randomized Clinical Trial | DOI: 10.2337/dc15-2078 | Not machine learning |
| Turksoy K, 2016 | IEEE Journal of Biomedical and Health Informatics | Meal Detection in Patients With Type 1 Diabetes: A New Module for the Multivariable Adaptive Artificial Pancreas Control System | DOI: 10.1109/jbhi.2015.2446413 | Not paediatric diabetes |
| Wysham C, 2016 | Diabetes Obes Metab | Baseline factors associated with glycaemic response to treatment with once-weekly dulaglutide in patients with type 2 diabetes | DOI: 10.1111/dom.12702 | Not paediatric diabetes |
| Xu L, 2016 | J Comput Graph Stat | Parameter Expanded Algorithms for Bayesian Latent Variable Modeling of Genetic Pleiotropy Data | DOI: 10.1080/10618600.2014.988337 | Not paediatric diabetes |
| Yamamoto Noguchi CC, 2016 | Advanced Biomedical Engineering | In Silico Blood Glucose Control for Type 1 Diabetes with Meal Announcement Using Carbohydrate Intake and Glycemic Index | DOI: 10.14326/abe.5.124 | Not paediatric diabetes |
| Zhong VW, 2016 | J Am Med Inform Assoc | An efficient approach for surveillance of childhood diabetes by type derived from electronic health record data: the SEARCH for Diabetes in Youth Study | DOI: 10.1093/jamia/ocv207 | Not machine learning |
| **Author, year** | **Journal** | **Title** | **Paper ID** | **Reason for exclusion** |
| Zuo W, 2016 | IEEE J Biomed Health Inform | Comparison of Three Different Types of Wrist Pulse Signals by Their Physical Meanings and Diagnosis Performance | DOI: 10.1109/JBHI.2014.2369821 | Not paediatric diabetes |
| Buckingham BA, 2017 | Diabetes Technol Ther | Evaluation of a Predictive Low-Glucose Management System In-Clinic | DOI: 10.1089/dia.2016.0319 | Not paediatric diabetes |
| Buckingham BA, 2017 | Diabetes | Safety and Feasibility of Omnipod Hybrid Closed-Loop in Adolescents with Type 1 Diabetes Using a Personalized Model Predictive Control Algorithm | DOI: 10.1089/dia.2017.0346 | Not machine learning |
| Cameron FM, 2017 | Diabetes Technology & Therapeutics | Closed-Loop Control Without Meal Announcement in Type 1 Diabetes | DOI: 10.1089/dia.2017.0078 | Not paediatric diabetes |
| Cheruku R, 2017 | International Journal of Computational Intelligence Systems | Diabetes Classification using Radial Basis Function Network by Combining Cluster Validity Index and BAT Optimization with Novel Fitness Function | DOI: 10.2991/ijcis.2017.10.1.17 | Not paediatric diabetes |
| Christiansen MP, 2017 | Diabetes Technology & Therapeutics | Accuracy of a Fourth-Generation Subcutaneous Continuous Glucose Sensor | DOI: 10.1089/dia.2017.0087 | Not paediatric diabetes |
| Colbaugh R, 2017 | Annu Int Conf IEEE Eng Med Biol Soc | Learning about individuals' health from aggregate data | DOI: 10.1109/EMBC.2017.8037514 | Not paediatric diabetes |
| DeBoer MD, 2017 | Pediatric Diabetes | Heart rate informed artificial pancreas system enhances glycemic control during exercise in adolescents with T1D | DOI: 10.1111/pedi.12454 | Not machine learning |
| Deja R, 2017 | Information Sciences | Hybrid approach to the generation of medical guidelines for insulin therapy for children | DOI: 10.1016/j.ins.2016.07.066 | Not machine learning |
| del Pino AS, 2017 | Endocrinologia Diabetes Y Nutricion | Is HLA the cause of the high incidence of type 1 diabetes in the Canary Islands? Results from the Type 1 Diabetes Genetics Consortium (T1DGC) | DOI: 10.1016/j.endien.2016.12.003 | Not machine learning |
| Dovc K, 2017 | Diabetologia | Closed-loop glucose control in young people with type 1 diabetes during and after unannounced physical activity: a randomised controlled crossover trial | DOI: 10.1007/s00125-017-4395-z | Not machine learning |
| Fergus P, 2017 | Neurocomputing | A machine learning approach to measure and monitor physical activity in children | DOI: 10.1016/j.neucom.2016.10.040 | Not paediatric diabetes |
| Goswami S, 2017 | Proceedings of the First International Conference on Intelligent Computing and Communication | Automatic Measurement and Analysis of Vessel Width in Retinal Fundus Image | DOI: 10.1007/978-981-10-2035-3_46 | Not paediatric diabetes |
| Griva L, 2017 | Ifac Papersonline | Commissioning procedure for predictive control based on ARX models of Type 1 Diabetes Mellitus patients | DOI: 10.1016/j.ifacol.2017.08.2482 | Not paediatric diabetes |
| Huang ZA, 2017 | Frontiers in Microbiology | PBHMDA: Path-Based Human Microbe-Disease Association Prediction | DOI: 10.3389/fmicb.2017.00233 | Not paediatric diabetes |
| Huyett LM, 2017 | Diabetes Technology & Therapeutics | Outpatient Closed-Loop Control with Unannounced Moderate Exercise in Adolescents Using Zone Model Predictive Control | DOI: 10.1089/dia.2016.0399 | Not machine learning |
| Jalal AH, 2017 | Selected Proceedings from the 232nd Ecs Meeting | A Fuel Cell based Sensing Platform for Selective Detection of Acetone in Hyperglycemic Patients | DOI: 10.1149/08010.1369ecst | Not paediatric diabetes |
| Karri SP, 2017 | Biomed Opt Express | Transfer learning based classification of optical coherence tomography images with diabetic macular edema and dry age-related macular degeneration | DOI: 10.1364/BOE.8.000579 | Not paediatric diabetes |
| Koh JEW, 2017 | Comput Biol Med | Diagnosis of retinal health in digital fundus images using continuous wavelet transform (CWT) and entropies | DOI: 10.1016/j.compbiomed.2017.03.008 | Not paediatric diabetes |
| Liu S, 2017 | AMIA Jt Summits Transl Sci Proc | Correlating Lab Test Results in Clinical Notes with Structured Lab Data: A Case Study in HbA1c and Glucose | PMID: 28815133 | Not paediatric diabetes |
| Lv XH, 2017 | Diabetology & Metabolic Syndrome | Interaction between peroxisome proliferator-activated receptor gamma polymorphism and obesity on type 2 diabetes in a Chinese Han population | DOI: 10.1186/s13098-017-0205-5 | Not paediatric diabetes |
| Ly TT, 2017 | Pediatric Diabetes | Automated hybrid closed-loop control with a proportional-integral-derivative based system in adolescents and adults with type 1 diabetes: individualizing settings for optimal performance | DOI: 10.1111/pedi.12399 | Not machine learning |
| **Author, year** | **Journal** | **Title** | **Paper ID** | **Reason for exclusion** |
| Moniotte S, 2017 | Pediatric Diabetes | Outcomes of algorithm-based modifications of insulinotherapy during exercise in MDI vs insulin pump-treated children with type 1 diabetes: Results from the TREAD-DIAB study | DOI: 10.1111/pedi.12509 | Not machine learning |
| Nath AP, 2017 | Genome Biol | An interaction map of circulating metabolites, immune gene networks, and their genetic regulation | DOI: 10.1186/s13059-017-1279-y | Not paediatric diabetes |
| Nguyen HV, 2017 | J Med Genet | Incremental cost-effectiveness of algorithm-driven genetic testing versus no testing for Maturity Onset Diabetes of the Young (MODY) in Singapore | DOI: 10.1136/jmedgenet-2017-104670 | Not paediatric diabetes |
| Nilashi M, 2017 | Fuzzy Information and Engineering | Accuracy Improvement for Diabetes Disease Classification: A Case on a Public Medical Dataset | DOI: 10.1016/j.fiae.2017.09.006 | Not paediatric diabetes |
| Perng W, 2017 | Obesity | Metabolomic Determinants of Metabolic Risk in Mexican Adolescents | DOI: 10.1002/oby.21926 | Not paediatric diabetes |
| Persson E, 2017 | Computational Statistics & Data Analysis | Data-driven algorithms for dimension reduction in causal inference | DOI: 10.1016/j.csda.2016.08.012 | Not paediatric diabetes |
| Pinsker JE, 2017 | Diabetes Technology & Therapeutics | Predictive Low-Glucose Suspend to Prevent Hypoglycemia | DOI: 10.1089/dia.2017.0064 | Not original research |
| Raghupathi V, 2017 | Healthcare (Basel) | Preventive Healthcare: A Neural Network Analysis of Behavioral Habits and Chronic Diseases | DOI: 10.3390/healthcare5010008 | Not paediatric diabetes |
| Ramkissoon CM, 2017 | Ifac Papersonline | Meal Detection in the Artificial Pancreas: Implications During Exercise | DOI: 10.1016/j.ifacol.2017.08.1083 | Not paediatric diabetes |
| Rogers MAM, 2017 | Bmc Medicine | Fluctuations in the incidence of type 1 diabetes in the United States from 2001 to 2015: a longitudinal study | DOI: 10.1186/s12916-017-0958-6 | Not machine learning |
| Rowlands AV, 2017 | J Sci Med Sport | Accelerometer wear-site detection: When one site does not suit all, all of the time | DOI: 10.1016/j.jsams.2016.04.013 | Not paediatric diabetes |
| Samouda H, 2017 | Pediatr Diabetes | Subtraction of subcutaneous fat to improve the prediction of visceral adiposity: exploring a new anthropometric track in overweight and obese youth | DOI: 10.1111/pedi.12415 | Not paediatric diabetes |
| Scaramuzza AE, 2017 | Acta Diabetologica | Use of the predictive low glucose management (PLGM) algorithm in Italian adolescents with type 1 diabetes: CareLink (TM) data download in a real-world setting | DOI: 10.1007/s00592-016-0927-0 | Not machine learning |
| Smith RJ, 2017 | J Med Internet Res | Variations in Facebook Posting Patterns Across Validated Patient Health Conditions: A Prospective Cohort Study | DOI: 10.2196/jmir.6486 | Not paediatric diabetes |
| Sun Y, 2017 | J Biomed Opt | Fully automated macular pathology detection in retina optical coherence tomography images using sparse coding and dictionary learning | DOI: 10.1117/1.JBO.22.1.016012 | Not paediatric diabetes |
| Tham LS, 2017 | J Clin Pharmacol | Modeling Pharmacokinetic Profiles of Insulin Regimens to Enhance Understanding of Subcutaneous Insulin Regimens | DOI: 10.1002/jcph.899 | Not paediatric diabetes |
| Turksoy K, 2017 | Control Engineering Practice | Real-time insulin bolusing for unannounced meals with artificial pancreas | DOI: 10.1016/j.conengprac.2016.08.001 | Not machine learning |
| Turksoy K, 2017 | IEEE Transactions on Biomedical Engineering | Real-Time Model-Based Fault Detection of Continuous Glucose Sensor Measurements | DOI: 10.1109/tbme.2016.2535412 | Not paediatric diabetes |
| Villani M, 2017 | BMC Health Serv Res | Time series modelling to forecast prehospital EMS demand for diabetic emergencies | DOI: 10.1186/s12913-017-2280-6 | Not paediatric diabetes |
| Wang XK, 2017 | Antonie Van Leeuwenhoek International Journal of General and Molecular Microbiology | Further analysis reveals new gut microbiome markers of type 2 diabetes mellitus | DOI: 10.1007/s10482-016-0805-3 | Not paediatric diabetes |
| Xu K, 2017 | Molecules | Deep Convolutional Neural Network-Based Early Automated Detection of Diabetic Retinopathy Using Fundus Image | DOI: 10.3390/molecules22122054 | Not paediatric diabetes |
| Yang JL, 2017 | Oncotarget | Using molecular functional networks to manifest connections between obesity and obesity-related diseases | DOI: 10.18632/oncotarget.19490 | Not paediatric diabetes |

| **Author, year** | **Journal** | **Title** | **Paper ID** | **Reason for exclusion** |
| --- | --- | --- | --- | --- |
| Yulina S, 2017 | Proceedings of the 2017 International Conference on Information Technology | Kids Menu Care: An Application for Food Menu Scheduling with Caloric Balance | DOI: 10.1145/3176653.3176732 | Not paediatric diabetes |
| Zhang Y, 2017 | Physiol Meas | Non-invasive blood glucose detection system based on conservation of energy method | DOI: 10.1088/1361-6579/aa50cf | Not paediatric diabetes |
| Zhang MH, 2017 | Nephron | The Impact of Peroxisome Proliferator-Activated Receptor Gamma and Its Interaction with Abdominal Obesity on Diabetic Nephropathy in Chinese Han | DOI: 10.1159/000450656 | Not paediatric diabetes |
| Brown JM, 2018 | Jama Ophthalmology | Automated Diagnosis of Plus Disease in Retinopathy of Prematurity Using Deep Convolutional Neural Networks | DOI: 10.1001/jamaophthalmol.2018.1934 | Not paediatric diabetes |
| Buckingham BA, 2018 | Diabetes Technology & Therapeutics | Safety and Feasibility of the OmniPod Hybrid Closed-Loop System in Adult, Adolescent, and Pediatric Patients with Type 1 Diabetes Using a Personalized Model Predictive Control Algorithm | DOI: 10.1089/dia.2017.0346 | Not machine learning |
| Cirillo E, 2018 | PLoS One | From SNPs to pathways: Biological interpretation of type 2 diabetes (T2DM) genome wide association study (GWAS) results | DOI: 10.1371/journal.pone.0193515 | Not original research |
| de Bock M, 2018 | Diabetes Technology & Therapeutics | Performance of Medtronic Hybrid Closed-Loop Iterations, Results from a Randomized Trial in Adolescents with Type 1 Diabetes | DOI: 10.1089/dia.2018.0161 | Not machine learning |
| de Bock M, 2018 | Bmj Open | Effect of 6 months hybrid closed-loop insulin delivery in young people with type 1 diabetes: a randomised controlled trial protocol | DOI: 10.1136/bmjopen-2017-020275 | Not original research |
| Do NV, 2018 | International Journal of Software Engineering and Knowledge Engineering | Knowledge-Based Model of Expert Systems Using Rela-Model | DOI: 10.1142/s0218194018500304 | Not paediatric diabetes |
| Forlenza GP, 2018 | Diabetes Care | Predictive Low-Glucose Suspend Reduces Hypoglycemia in Adults, Adolescents, and Children With Type 1 Diabetes in an At-Home Randomized Crossover Study: Results of the PROLOG Trial | DOI: 10.2337/dc18-0771 | Not paediatric diabetes |
| Gholipour K, 2018 | East Mediterr Health J | Modelling the prevalence of diabetes mellitus risk factors based on artificial neural network and multiple regression | DOI: 10.26719/emhj.18.012 | Not paediatric diabetes |
| Gonzalez-Gil EM, 2018 | European Journal of Nutrition | Prospective associations between dietary patterns and high sensitivity C-reactive protein in European children: the IDEFICS study | DOI: 10.1007/s00394-017-1419-x | Not paediatric diabetes |
| Gosho M, 2018 | J Clin Pharmacol | Risk of Hypoglycemia After Concomitant Use of Antidiabetic, Antihypertensive, and Antihyperlipidemic Medications: A Database Study | DOI: 10.1002/jcph.1147 | Not paediatric diabetes |
| Gotze A, 2018 | Scientific Reports | The corneal subbasal nerve plexus and thickness of the retinal layers in paediatric type 1 diabetes and matched controls | DOI: 10.1038/s41598-017-18284-z | Not machine learning |
| Han Y, 2018 | Parasitol Res | The association between Toxoplasma gondii infection and hypertensive disorders in T2DM patients: a case-control study in the Han Chinese population | DOI: 10.1007/s00436-017-5737-y | Not paediatric diabetes |
| He GN, 2018 | Bmc Systems Biology | A hotspots analysis-relation discovery representation model for revealing diabetes mellitus and obesity | DOI: 10.1186/s12918-018-0640-4 | Not paediatric diabetes |
| Herrero P, 2018 | J Diabetes Sci Technol | Automatic Adaptation of Basal Insulin Using Sensor-Augmented Pump Therapy | DOI: 10.1177/1932296818761752 | Not machine learning |
| Huang Y, 2018 | Diabetes Research and Clinical Practice | Gut microbiota profiling in Han Chinese with type 1 diabetes | DOI: 10.1016/j.diabres.2018.04.032 | Not paediatric diabetes |
| Hussain MA, 2018 | PLoS One | Classification of healthy and diseased retina using SD-OCT imaging and Random Forest algorithm | DOI: 10.1371/journal.pone.0198281 | Not paediatric diabetes |
| Hussain A, 2018 | IEEE/ACM Trans Comput Biol Bioinform | Backpropagation Approach Supported by Image Compression Algorithm for the Classification of Chronic Condition Diseases | DOI: 10.1109/TCBB.2018.2878556 | Not paediatric diabetes |
| Jiang Z, 2018 | Biomed Eng Online | A super-resolution method-based pipeline for fundus fluorescein angiography imaging | DOI: 10.1186/s12938-018-0556-7 | Not paediatric diabetes |
| Kermany DS, 2018 | Cell | Identifying Medical Diagnoses and Treatable Diseases by Image-Based Deep Learning | DOI: 10.1016/j.cell.2018.02.010 | Not paediatric diabetes |
| **Author, year** | **Journal** | **Title** | **Paper ID** | **Reason for exclusion** |
| Kundu R, 2018 | Plos One | In silico and ex vivo approaches indicate immune pressure on capsid and non-capsid regions of coxsackie B viruses in the human system | DOI: 10.1371/journal.pone.0199323 | Not paediatric diabetes |
| Kwak SH, 2018 | Diabetes | Nonsynonymous Variants in PAX4 and GLP1R Are Associated With Type 2 Diabetes in an East Asian Population | DOI: 10.2337/db18-0361 | Not paediatric diabetes |
| Ladyzynski P, 2018 | Nutrients | Accuracy of Automatic Carbohydrate, Protein, Fat and Calorie Counting Based on Voice Descriptions of Meals in People with Type 1 Diabetes | DOI: 10.3390/nu10040518 | Not paediatric diabetes |
| Lauria M, 2018 | Scientific Reports | Consensus Clustering of temporal profiles for the identification of metabolic markers of pre-diabetes in childhood (EarlyBird 73) | DOI: 10.1038/s41598-017-19059-2 | Not machine learning |
| Lewandowska K, 2018 | Autoimmunity | Celiac antibodies in children with type 1 diabetes - A diagnostic validation study | DOI: 10.1080/08916934.2018.1427226 | Not machine learning |
| Li G, 2018 | Cardiovasc Diabetol | Childhood retinol-binding protein 4 (RBP4) levels predicting the 10-year risk of insulin resistance and metabolic syndrome: the BCAMS study | DOI: 10.1186/s12933-018-0707-y | Not paediatric diabetes |
| Lopez PE, 2018 | Diabetic Medicine | A randomized comparison of three prandial insulin dosing algorithms for children and adolescents with Type 1 diabetes | DOI: 10.1111/dme.13703 | Not machine learning |
| Nilashi M, 2018 | Health Informatics Journal | A soft computing approach for diabetes disease classification | DOI: 10.1177/1460458216675500 | Not paediatric diabetes |
| Nimri R, 2018 | Diabetes Obesity & Metabolism | Adjusting insulin doses in patients with type 1 diabetes who use insulin pump and continuous glucose monitoring: Variations among countries and physicians | DOI: 10.1111/dom.13408 | Not paediatric diabetes |
| Oppenheimer S, 2018 | Eur J Pediatr | The impact of coping patterns and chronic health conditions on health-related quality of life among children and adolescents | DOI: 10.1007/s00431-018-3146-6 | Not machine learning |
| Ortiz-Rubio P, 2018 | Pediatric Diabetes | Adjusting Insulin Delivery to Activity (AIDA) clinical trial: Effects of activity-based insulin profiles on glucose control in children with type 1 diabetes | DOI: 10.1111/pedi.12752 | Not machine learning |
| Paul R, 2018 | Spat Spatiotemporal Epidemiol | Assessing the association of diabetes self-management education centers with age-adjusted diabetes rates across U.S.: Aspatial cluster analysis approach | DOI: 10.1016/j.sste.2017.11.002 | Not paediatric diabetes |
| Petrovski G, 2018 | Diabetes Therapy | Continuous Subcutaneous Insulin Infusion Characteristics in Type 1 Diabetes Children and Adolescents in Qatar | DOI: 10.1007/s13300-018-0510-5 | Not machine learning |
| Ramkissoon CM, 2018 | Sensors | Unannounced Meals in the Artificial Pancreas: Detection Using Continuous Glucose Monitoring | DOI: 10.3390/s18030884 | Not paediatric diabetes |
| Ramsingh J, 2018 | Proceedings of the 2018 Second International Conference on Intelligent Computing and Control Systems | A Big Data framework to analyze risk factors of diabetes outbreak in Indian population using a MapReduce algorithm | DOI: 10.1109/ICCONS.2018.8663143 | Not paediatric diabetes |
| Shakeel PM, 2018 | Health Inf Sci Syst | Cloud based framework for diagnosis of diabetes mellitus using K-means clustering | DOI: 10.1007/s13755-018-0054-0 | Not paediatric diabetes |
| Sun L, 2018 | Bioscience Reports | Association of single nucleotide polymorphisms in CACNA 1A/CACNA 1C/CACNA 1H calcium channel genes with diabetic peripheral neuropathy in Chinese population | DOI: 10.1042/bsr20171670 | Not paediatric diabetes |
| Thishya K, 2018 | PLoS One | Artificial neural network model for predicting the bioavailability of tacrolimus in patients with renal transplantation | DOI: 10.1371/journal.pone.0191921 | Not paediatric diabetes |
| Wang Y, 2018 | Gene | Triangular relationship between CYP2R1 gene polymorphism, serum 25(OH)D-3 levels and T2DM in a Chinese rural population | DOI: 10.1016/j.gene.2018.08.006 | Not paediatric diabetes |
| Wood MA, 2018 | Diabetes Technology & Therapeutics | In-Clinic Evaluation of the MiniMed 670G System "Suspend Before Low" Feature in Children with Type 1 Diabetes | DOI: 10.1089/dia.2018.0209 | Not machine learning |
| Yang AT, 2018 | Journal of Bioinformatics and Computational Biology | Constrained maximum entropy models to select genotype interactions associated with censored failure times | DOI: 10.1142/s0219720018400243 | Not paediatric diabetes |
| Ye C, 2018 | J Med Internet Res | Prediction of Incident Hypertension Within the Next Year: Prospective Study Using Statewide Electronic Health Records and Machine Learning | DOI: 10.2196/jmir.9268 | Not paediatric diabetes |
| **Author, year** | **Journal** | **Title** | **Paper ID** | **Reason for exclusion** |
| Zhang Y, 2018 | Technol Health Care | Association between serum magnesium and common complications of diabetes mellitus | DOI: 10.3233/THC-174702 | Not paediatric diabetes |
| Aharony O, 2019 | Transl Vis Sci Technol | Automatic Characterization of Retinal Blood Flow Using OCT Angiograms | DOI: 10.1167/tvst.8.4.6 | Not paediatric diabetes |
| Arsalan M, 2019 | J Clin Med | Aiding the Diagnosis of Diabetic and Hypertensive Retinopathy Using Artificial Intelligence-Based Semantic Segmentation | DOI: 10.3390/jcm8091446 | Not paediatric diabetes |
| Balyan R, 2019 | PLoS One | Using natural language processing and machine learning to classify health literacy from secure messages: The ECLIPPSE study | DOI: 10.1371/journal.pone.0212488 | Not paediatric diabetes |
| Basu S, 2019 | Med Care | A Prediction Model for Uncontrolled Type 2 Diabetes Mellitus Incorporating Area-level Social Determinants of Health | DOI: 10.1097/MLR.0000000000001147 | Not paediatric diabetes |
| Berkowitz SA, 2019 | BMJ Open | Association between access to social service resources and cardiometabolic risk factors: a machine learning and multilevel modeling analysis | DOI: 10.1136/bmjopen-2018-025281 | Not paediatric diabetes |
| Biester T, 2019 | Diabetes Obes Metab | DREAM5: An open-label, randomized, cross-over study to evaluate the safety and efficacy of day and night closed-loop control by comparing the MD-Logic automated insulin delivery system to sensor augmented pump therapy in patients with type 1 diabetes at home | DOI: 10.1111/dom.13585 | Not machine learning |
| Burckhardt MA, 2019 | Diabetes Technology & Therapeutics | Use of Continuous Glucose Monitoring Trends to Facilitate Exercise in Children with Type 1 Diabetes | DOI: 10.1089/dia.2018.0292 | Not machine learning |
| Chen L, 2019 | Current Developments in Nutrition | Race, Gender, Family Structure, Socioeconomic Status, Dietary Patterns, and Cardiovascular Health in Adolescents | DOI: 10.1093/cdn/nzz117 | Not paediatric diabetes |
| Cherubini V, 2019 | Pediatric Diabetes | Optimal predictive low glucose management settings during physical exercise in adolescents with type 1 diabetes | DOI: 10.1111/pedi.12792 | Not machine learning |
| Clark MM, 2019 | Sci Transl Med | Diagnosis of genetic diseases in seriously ill children by rapid whole-genome sequencing and automated phenotyping and interpretation | DOI: 10.1126/scitranslmed.aat6177 | Not paediatric diabetes |
| Cuesta HA, 2019 | Health Place | Using decision trees to understand the influence of individual- and neighborhood-level factors on urban diabetes and asthma | DOI: 10.1016/j.healthplace.2019.04.009 | Not paediatric diabetes |
| Czerniecki JM, 2019 | Br J Surg | Predicting reamputation risk in patients undergoing lower extremity amputation due to the complications of peripheral artery disease and/or diabetes | DOI: 10.1002/bjs.11160 | Not paediatric diabetes |
| Czmil A, 2019 | Applied Sciences-Basel | A Method to Detect Type 1 Diabetes Based on Physical Activity Measurements Using a Mobile Device | DOI: 10.3390/app9122555 | Not machine learning |
| Dadios M, 2019 | Critical Care Medicine | DOES AN IVF TITRATION ALGORITHM IMPROVE GLUCOSE CONTROL IN CHILDREN WITH DIABETIC KETOACIDOSIS? | DOI: 10.1097/01.ccm.0000551061.02360.99 | Not machine learning |
| Ehrenfeld JM, 2019 | Ethn Dis | Development of a Natural Language Processing Algorithm to Identify and Evaluate Transgender Patients in Electronic Health Record Systems | DOI: 10.18865/ed.29.S2.441 | Not paediatric diabetes |
| El Fathi A, 2019 | 2019 American Control Conference | An Unannounced Meal Detection Module for Artificial Pancreas Control Systems | DOI: 10.23919/ACC.2019.8814932 | Not paediatric diabetes |
| Endesfelder D, 2019 | Diabetes | Time-Resolved Autoantibody Profiling Facilitates Stratification of Preclinical Type 1 Diabetes in Children | DOI: 10.2337/db18-0594 | Not machine learning |
| Forlenza GP, 2019 | Diabetes Technology & Therapeutics | Use of Artificial Intelligence to Improve Diabetes Outcomes in Patients Using Multiple Daily Injections Therapy | DOI: 10.1089/dia.2019.0077 | Not original research |
| Forlenza GP, 2019 | Diabetes Technology & Therapeutics | Successful At-Home Use of the Tandem Control-IQ Artificial Pancreas System in Young Children During a Randomized Controlled Trial | DOI: 10.1089/dia.2019.0011 | Not machine learning |
| Gomez AM, 2019 | Diabetes & Metabolic Syndrome-Clinical Research & Reviews | Impact of sensor-augmented pump therapy with predictive low-glucose management on hypoglycemia and glycemic control in patients with type 1 diabetes mellitus: 1-year follow-up | DOI: 10.1016/j.dsx.2019.07.024 | Not paediatric diabetes |
| Gonoodi K, 2019 | Diabetes Metab Syndr | An assessment of the risk factors for vitamin D deficiency using a decision tree model | DOI: 10.1016/j.dsx.2019.03.020 | Not paediatric diabetes |
| **Author, year** | **Journal** | **Title** | **Paper ID** | **Reason for exclusion** |
| Gubbi S, 2019 | Front Endocrinol (Lausanne) | Artificial Intelligence and Machine Learning in Endocrinology and Metabolism: The Dawn of a New Era | DOI: 10.3389/fendo.2019.00185 | Not original research |
| Hernández-Montoya D, 2019 | Ann Epidemiol | Variation in incidence of type 2 diabetes mellitus: time series of Mexican adolescents | DOI: 10.1016/j.annepidem.2018.11.006 | Not machine learning |
| Hernandez-Montoya D, 2019 | Advances in Life Course Research | A life-course approach to early-onset of diabetes mellitus: Probable contribution of collective violence in Mexico | DOI: 10.1016/j.alcr.2019.03.006 | Not machine learning |
| Huang RC, 2019 | Journal of Clinical Endocrinology & Metabolism | Epigenetic Age Acceleration in Adolescence Associates With BMI, Inflammation, and Risk Score for Middle Age Cardiovascular Disease | DOI: 10.1210/jc.2018-02076 | Not paediatric diabetes |
| Jacobsen LM, 2019 | Pediatric Diabetes | Predicting progression to type 1 diabetes from ages 3 to 6 in islet autoantibody positive TEDDY children | DOI: 10.1111/pedi.12812 | Not machine learning |
| Jung O, 2019 | Stud Health Technol Inform | Empowering Diabetes Patients with Interventions Based on Behaviour Change Techniques | PMID: 31118332 | Not original research |
| Kaptein F, 2019 | 2019 8th International Conference on Affective Computing and Intelligent Interaction | Evaluating Cognitive and Affective Intelligent Agent Explanations in a Long-Term Health-Support Application for Children with Type 1 Diabetes | DOI: 10.1109/ACII.2019.8925526 | Not machine learning |
| Kim YG, 2019 | Pattern Recognition Letters | Valid oversampling schemes to handle imbalance | DOI: 10.1016/j.patrec.2019.07.006 | Not paediatric diabetes |
| Kohn JR, 2019 | Pediatric Diabetes | Outcomes, care utilization, and expenditures in adolescent pregnancy complicated by diabetes | DOI: 10.1111/pedi.12871 | Not machine learning |
| Kwon JY, 2019 | Comput Inform Nurs | Nurses "Seeing Forest for the Trees" in the Age of Machine Learning: Using Nursing Knowledge to Improve Relevance and Performance | DOI: 10.1097/CIN.0000000000000508 | Not paediatric diabetes |
| Lee I, 2019 | J Pediatr Nurs | Risk Factors for Obesity Among Children Aged 24 to 80 months in Korea: A Decision Tree Analysis | DOI: 10.1016/j.pedn.2019.02.004 | Not paediatric diabetes |
| Liu JJ, 2019 | Arterioscler Thromb Vasc Biol | Arterial Stiffness Modulates the Association of Resting Heart Rate With Rapid Renal Function Decline in Individuals With Type 2 Diabetes Mellitus | DOI: 10.1161/ATVBAHA.119.313163 | Not paediatric diabetes |
| Liu Y, 2019 | Risk Manag Healthc Policy | Machine Learning For Tuning, Selection, And Ensemble Of Multiple Risk Scores For Predicting Type 2 Diabetes | DOI: 10.2147/RMHP.S225762 | Not paediatric diabetes |
| Liu WP, 2019 | Artificial Organs | Improved Generalized Predictive Control Algorithm for Blood Glucose Control of Type 1 Diabetes | DOI: 10.1111/aor.13350 | Not machine learning |
| Mahikul W, 2019 | Int J Environ Res Public Health | A Population Dynamic Model to Assess the Diabetes Screening and Reporting Programs and Project the Burden of Undiagnosed Diabetes in Thailand | DOI: 10.3390/ijerph16122207 | Not machine learning |
| Mayo M, 2019 | Plos One | Glycemic-aware metrics and oversampling techniques for predicting blood glucose levels using machine learning | DOI: 10.1371/journal.pone.0225613 | Not paediatric diabetes |
| Memari N, 2019 | Journal of Medical and Biological Engineering | Retinal Blood Vessel Segmentation by Using Matched Filtering and Fuzzy C-means Clustering with Integrated Level Set Method for Diabetic Retinopathy Assessment | DOI: 10.1007/s40846-018-0454-2 | Not paediatric diabetes |
| Migliorelli L, 2019 | 2019 IEEE 23rd International Symposium on Consumer Technologies | MyDi application: towards automatic activity annotation of young patients with Type 1 diabetes | DOI: 10.1109/ISCE.2019.8901017 | Not paediatric diabetes |
| Nakhla M, 2019 | Clin Epidemiol | Identifying paediatric diabetes cases from health administrative data: a population-based validation study in Quebec, Canada | DOI: 10.2147/CLEP.S217969 | Not machine learning |
| Neerincx MA, 2019 | Front Robot AI | Socio-Cognitive Engineering of a Robotic Partner for Child's Diabetes Self-Management | DOI: 10.3389/frobt.2019.00118 | Not machine learning |
| Pei D, 2019 | J Diabetes Res | Identification of Potential Type II Diabetes in a Chinese Population with a Sensitive Decision Tree Approach | DOI: 10.1155/2019/4248218 | Not paediatric diabetes |
| Pesenacker AM, 2019 | Jci Insight | Treg gene signatures predict and measure type 1 diabetes trajectory | DOI: 10.1172/jci.insight.123879 | Not paediatric diabetes |
| Piffaretti C, 2019 | Diabetes Research and Clinical Practice | Trends in childhood type 1 diabetes incidence in France, 2010-2015 | DOI: 10.1016/j.diabres.2018.11.005 | Not machine learning |
| **Author, year** | **Journal** | **Title** | **Paper ID** | **Reason for exclusion** |
| Poonguzhali S, 2019 | Journal of Clinical and Diagnostic Research | Retinal Health Investigation by Segmentation or Major and Minor Blood Vessels in Fundus Images for Diabetes Patients | DOI: 10.7860/jcdr/2019/41221.13207 | Not paediatric diabetes |
| Rasti R, 2019 | J Med Signals Sens | Convolutional Mixture of Experts Model: A Comparative Study on Automatic Macular Diagnosis in Retinal Optical Coherence Tomography Imaging | DOI: 10.4103/jmss.JMSS_27_17 | Not paediatric diabetes |
| Renard E, 2019 | Diabetes Obesity & Metabolism | Closed-loop driven by control-to-range algorithm outperforms threshold-low-glucose-suspend insulin delivery on glucose control albeit not on nocturnal hypoglycaemia in prepubertal patients with type 1 diabetes in a supervised hotel setting | DOI: 10.1111/dom.13482 | Not machine learning |
| Rodríguez-Rodríguez I, 2019 | Sensors (Basel) | Utility of Big Data in Predicting Short-Term Blood Glucose Levels in Type 1 Diabetes Mellitus Through Machine Learning Techniques | DOI: 10.3390/s19204482 | Not paediatric diabetes |
| Rodríguez-Rodríguez I, 2019 | Sensors (Basel) | On the Possibility of Predicting Glycaemia 'On the Fly' with Constrained IoT Devices in Type 1 Diabetes Mellitus Patients | DOI: 10.3390/s19204538 | Not paediatric diabetes |
| Schierloh U, 2019 | Plos One | Lower plasma insulin levels during overnight closed-loop in school children with type 1 diabetes: Potential advantage? A randomized cross-over trial | DOI: 10.1371/journal.pone.0212013 | Not machine learning |
| Singh B, 2019 | Proceedings of the 2019 10th International Conference on Dependable Systems, Services and Technologies | A Machine Learning Approach for Predicting Weight Gain Risks in Young Adults | DOI: 10.1109/DESSERT.2019.8770016 | Not paediatric diabetes |
| Tauschmann M, 2019 | Diabetes Care | Home Use of Day-and-Night Hybrid Closed-Loop Insulin Delivery in Very Young Children: A Multicenter, 3-Week, Randomized Trial | DOI: 10.2337/dc18-1881 | Not machine learning |
| Teltsch DY, 2019 | Pharmacoepidemiol Drug Saf | Development and validation of algorithms to identify newly diagnosed type 1 and type 2 diabetes in paediatric population using electronic medical records and claims data | DOI: 10.1002/pds.4728 | Not machine learning |
| Teltsch DY, 2019 | Pharmacoepidemiology and Drug Safety | Development and validation of algorithms to identify newly diagnosed type 1 and type 2 diabetes in paediatric population using electronic medical records and claims data | DOI: 10.1002/pds.4728 | Not machine learning |
| Ting DSW, 2019 | NPJ Digit Med | Deep learning in estimating prevalence and systemic risk factors for diabetic retinopathy: a multi-ethnic study | DOI: 10.1038/s41746-019-0097-x | Not paediatric diabetes |
| **Author, year** | **Journal** | **Title** | **Paper ID** | **Reason for exclusion** |
| Tronstad C, 2019 | Anal Chim Acta | Non-invasive prediction of blood glucose trends during hypoglycemia | DOI: 10.1016/j.aca.2018.12.009 | Not paediatric diabetes |
| Vranckx M, 2019 | Spat Spatiotemporal Epidemiol | Comparison of different software implementations for spatial disease mapping | DOI: 10.1016/j.sste.2019.100302 | Not machine learning |
| Williams SA, 2019 | Nat Med | Plasma protein patterns as comprehensive indicators of health | DOI: 10.1038/s41591-019-0665-2 | Not paediatric diabetes |
| You Y, 2019 | BMC Med Inform Decis Mak | Application of machine learning methodology to assess the performance of DIABETIMSS program for patients with type 2 diabetes in family medicine clinics in Mexico | DOI: 10.1186/s12911-019-0950-5 | Not paediatric diabetes |
| Zhang L, 2019 | Genes (Basel) | Detection of Microaneurysms in Fundus Images Based on an Attention Mechanism | DOI: 10.3390/genes10100817 | Not paediatric diabetes |
| Zhao XX, 2019 | IEEE Access | Fine-Grained Diabetic Wound Depth and Granulation Tissue Amount Assessment Using Bilinear Convolutional Neural Network | DOI: 10.1109/access.2019.2959027 | Not paediatric diabetes |
| Abuin P, 2020 | Journal of Process Control | Artificial pancreas under stable pulsatile MPC: Improving the closed-loop performance | DOI: 10.1016/j.jprocont.2020.06.009 | Not paediatric diabetes |
| Ahne A, 2020 | BMJ Open Diabetes Res Care | Insulin pricing and other major diabetes-related concerns in the USA: a study of 46 407 tweets between 2017 and 2019 | DOI: 10.1136/bmjdrc-2020-001190 | Not paediatric diabetes |
| Anjana RM, 2020 | BMJ Open Diabetes Res Care | Novel subgroups of type 2 diabetes and their association with microvascular outcomes in an Asian Indian population: a data-driven cluster analysis: the INSPIRED study | DOI: 10.1136/bmjdrc-2020-001506 | Not paediatric diabetes |
| **Author, year** | **Journal** | **Title** | **Paper ID** | **Reason for exclusion** |
| Avari P, 2020 | Diabetes Technol Ther | Safety and feasibility of the PEPPER adaptive bolus advisor and safety system; a randomized control study | DOI: 10.1089/dia.2020.0301 | Not paediatric diabetes |
| Bhatia KK, 2020 | Retina | DISEASE CLASSIFICATION OF MACULAR OPTICAL COHERENCE TOMOGRAPHY SCANS USING DEEP LEARNING SOFTWARE: Validation on Independent, Multicenter Data | DOI: 10.1097/IAE.0000000000002640 | Not paediatric diabetes |
| Burnside M, 2020 | J Diabetes Sci Technol | Do-It-Yourself Automated Insulin Delivery: A Leading Example of the Democratization of Medicine | DOI: 10.1177/1932296819890623 | Not original research |
| Burnside M, 2020 | Journal of Diabetes and Metabolic Disorders | CREATE (Community deRivEd AutomaTEd insulin delivery) trial. Randomised parallel arm open label clinical trial comparing automated insulin delivery using a mobile controller (AnyDANA-loop) with an open-source algorithm with sensor augmented pump therapy in type 1 diabetes | DOI: 10.1007/s40200-020-00547-8 | Not machine learning |
| Chakrabarty A, 2020 | IEEE Transactions on Control Systems Technology | Embedded Model Predictive Control for a Wearable Artificial Pancreas | DOI: 10.1109/tcst.2019.2939122 | Not paediatric diabetes |
| Chirita-Emandi A, 2020 | Diabetes Metabolic Syndrome and Obesity-Targets and Therapy | CHDH-PNPLA3 Gene-Gene Interactions Predict Insulin Resistance in Children with Obesity | DOI: 10.2147/dmso.S277268 | Not paediatric diabetes |
| Cichosz SL, 2020 | J Diabetes Sci Technol | Precise Prediction of Total Body Lean and Fat Mass From Anthropometric and Demographic Data: Development and Validation of Neural Network Models | DOI: 10.1177/1932296820971348 | Not paediatric diabetes |
| Dworzynski P, 2020 | Sci Rep | Nationwide prediction of type 2 diabetes comorbidities | DOI: 10.1038/s41598-020-58601-7 | Not paediatric diabetes |
| El Fathi A, 2020 | Pediatric Diabetes | A pilotnon-inferiorityrandomized controlled trial to assess automatic adjustments of insulin doses in adolescents with type 1 diabetes on multiple daily injections therapy | DOI: 10.1111/pedi.13052 | Not machine learning |
| Ferreira JP, 2020 | Clin Res Cardiol | Plasma protein biomarkers and their association with mutually exclusive cardiovascular phenotypes: the FIBRO-TARGETS case-control analyses | DOI: 10.1007/s00392-019-01480-4 | Not paediatric diabetes |
| Fushimi E, 2020 | Medical & Biological Engineering & Computing | Automatic glycemic regulation for the paediatric population based on switched control and time-varying IOB constraints: an in silico study | DOI: 10.1007/s11517-020-02213-w | Not machine learning |
| Guemes A, 2020 | IEEE Journal of Biomedical and Health Informatics | Predicting Quality of Overnight Glycaemic Control in Type 1 Diabetes Using Binary Classifiers | DOI: 10.1109/jbhi.2019.2938305 | Not paediatric diabetes |
| Gutmann DAP, 2020 | Acad Radiol | MRI-Derived Radiomics Features of Hepatic Fat Predict Metabolic States in Individuals without Cardiovascular Disease | DOI: 10.1016/j.acra.2020.06.030 | Not paediatric diabetes |
| Hu F, 2020 | Methods Inf Med | Predicting Lipid-Lowering Medication Persistence after the First Cardiovascular Disease Hospitalization | DOI: 10.1055/s-0040-1713905 | Not paediatric diabetes |
| Huang JL, 2020 | Diabetes | Machine Learning Approaches Reveal Metabolic Signatures of Incident Chronic Kidney Disease in Individuals With Prediabetes and Type 2 Diabetes | DOI: 10.2337/db20-0586 | Not paediatric diabetes |
| Jiang T, 2020 | Stat Med | A model-based approach for clustering of multivariate semicontinuous data with application to dietary pattern analysis and intervention | DOI: 10.1002/sim.8391 | Not machine learning |
| Jiang T, 2020 | Statistics in Medicine | A model-based approach for clustering of multivariate semicontinuous data with application to dietary pattern analysis and intervention | DOI: 10.1002/sim.8391 | Not machine learning |
| Kane P, 2020 | Journal of Paediatrics and Child Health | Early identification of cardiac autonomic neuropathy using complexity analysis in children with type 1 diabetes | DOI: 10.1111/jpc.14745 | Not machine learning |
| Ke C, 2020 | BMC Med Res Methodol | Development and validation of algorithms to classify type 1 and 2 diabetes according to age at diagnosis using electronic health records | DOI: 10.1186/s12874-020-00921-3 | Not machine learning |
| Ke CV, 2020 | Bmc Medical Research Methodology | Development and validation of algorithms to classify type 1 and 2 diabetes according to age at diagnosis using electronic health records | DOI: 10.1186/s12874-020-00921-3 | Not machine learning |
| Kim YD, 2020 | Sci Rep | Effects of Hypertension, Diabetes, and Smoking on Age and Sex Prediction from Retinal Fundus Images | DOI: 10.1038/s41598-020-61519-9 | Not paediatric diabetes |
| **Author, year** | **Journal** | **Title** | **Paper ID** | **Reason for exclusion** |
| Kunin A, 2020 | EPMA J | Voice perturbations under the stress overload in young individuals: phenotyping and suboptimal health as predictors for cascading pathologies | DOI: 10.1007/s13167-020-00229-8 | Not paediatric diabetes |
| Lauffenburger JC, 2020 | BMC Endocr Disord | Not there yet: using data-driven methods to predict who becomes costly among low-cost patients with type 2 diabetes | DOI: 10.1186/s12902-020-00609-1 | Not paediatric diabetes |
| Liu D, 2020 | Scientific Reports | Interaction Between Apolipoprotein M Gene Single-Nucleotide Polymorphisms and Obesity and its Effect on Type 2 Diabetes Mellitus Susceptibility | DOI: 10.1038/s41598-020-64467-6 | Not paediatric diabetes |
| Lo YC, 2020 | Sci Rep | Epiretinal Membrane Detection at the Ophthalmologist Level using Deep Learning of Optical Coherence Tomography | DOI: 10.1038/s41598-020-65405-2 | Not paediatric diabetes |
| Magdelaine N, 2020 | Iet Systems Biology | Hypoglycaemia-free artificial pancreas project | DOI: 10.1049/iet-syb.2018.5069 | Not machine learning |
| Mirabedini S, 2020 | Bioscience Research | Diabetic retinopathy classification via Generative Adversarial Networks | ISSN: 2218-3973 | Not paediatric diabetes |
| Mitani A, 2020 | Nature Biomedical Engineering | Detection of anaemia from retinal fundus images via deep learning | DOI: 10.1038/s41551-019-0487-z | Not paediatric diabetes |
| Mosslemi M, 2020 | Cardiovasc Endocrinol Metab | A treatment-based algorithm for identification of diabetes type in the National Health and Nutrition Examination Survey | DOI: 10.1097/XCE.0000000000000189 | Not paediatric diabetes |
| Mosquera-Lopez C, 2020 | Diabetes Technology & Therapeutics | Predicting and Preventing Nocturnal Hypoglycemia in Type 1 Diabetes Using Big Data Analytics and Decision Theoretic Analysis | DOI: 10.1089/dia.2019.0458 | Not paediatric diabetes |
| Myneni S, 2020 | JMIR Med Inform | Diabetes Self-Management in the Age of Social Media: Large-Scale Analysis of Peer Interactions Using Semiautomated Methods | DOI: 10.2196/18441 | Not paediatric diabetes |
| Nimri R, 2020 | J Diabetes Sci Technol | Adjustment of Insulin Pump Settings in Type 1 Diabetes Management: Advisor Pro Device Compared to Physicians' Recommendations | DOI: 10.1177/1932296820965561 | Not machine learning |
| Palisaitis E, 2020 | Diabetes Technology & Therapeutics | The Efficacy of Basal Rate and Carbohydrate Ratio Learning Algorithm for Closed-Loop Insulin Delivery (Artificial Pancreas) in Youth with Type 1 Diabetes in a Diabetes Camp | DOI: 10.1089/dia.2019.0270 | Not machine learning |
| Pan XJ, 2020 | Graefes Archive for Clinical and Experimental Ophthalmology | Multi-label classification of retinal lesions in diabetic retinopathy for automatic analysis of fundus fluorescein angiography based on deep learning | DOI: 10.1007/s00417-019-04575-w | Not paediatric diabetes |
| Porwal P, 2020 | Med Image Anal | IDRiD: Diabetic Retinopathy - Segmentation and Grading Challenge | DOI: 10.1016/j.media.2019.101561 | Not paediatric diabetes |
| Riaz H, 2020 | Diagnostics | Deep and Densely Connected Networks for Classification of Diabetic Retinopathy | DOI: 10.3390/diagnostics10010024 | Not paediatric diabetes |
| Rim TH, 2020 | Lancet Digit Health | Prediction of systemic biomarkers from retinal photographs: development and validation of deep-learning algorithms | DOI: 10.1016/S2589-7500(20)30216-8 | Not paediatric diabetes |
| Rodrigues AMAM, 2020 | Cien Saude Colet | [Use of the health services according to social determinants, health behaviors and quality of life among diabetics] | DOI: 10.1590/1413-81232020253.19532018 | Not paediatric diabetes |
| Ruamviboonsuk P, 2020 | Asia Pac J Ophthalmol (Phila) | Artificial Intelligence in Ophthalmology: Evolutions in Asia | DOI: 10.1097/01.APO.0000656980.41190.bf | Not original research |
| Ruiz-Velazquez E, 2020 | Journal of the Franklin Institute-Engineering and Applied Mathematics | Robust mu-synthesis: Towards a unified glucose control in adults, adolescents and children with T1DM | DOI: 10.1016/j.jfranklin.2020.07.030 | Not machine learning |
| Schmidt BM, 2020 | Journal of Diabetes and Its Complications | Strategies to reduce severe diabetic foot infections and complications during epidemics (STRIDE) | DOI: 10.1016/j.jdiacomp.2020.107691 | Not paediatric diabetes |
| Sherr J, 2020 | Diabetes Technology & Therapeutics | PERFORMANCE OF OMNIPOD PERSONALIZED MODEL PREDICTIVE CONTROL ALGORITHM WITH MULTIPLE SETPOINTS AND MEAL AND EXERCISE CHALLENGES IN CHILDREN AGED 2-12 YEARS WITH TYPE 1 DIABETES | DOI: 10.1089/dia.2019.0286 | Not paediatric diabetes |
| Sherr JL, 2020 | Diabetes Technology & Therapeutics | Safety and Performance of the Omnipod Hybrid Closed-Loop System in Adults, Adolescents, and Children with Type 1 Diabetes Over 5 Days Under Free-Living Conditions | DOI: 10.1089/dia.2019.0286 | Not machine learning |
| **Author, year** | **Journal** | **Title** | **Paper ID** | **Reason for exclusion** |
| Taylor KA, 2020 | Diabetes Technology & Therapeutics | Use of Machine Learning and Hybrid Closed Loop Insulin Delivery at Diabetes Camps | DOI: 10.1089/dia.2020.0026 | Not original research |
| Tragomalou A, 2020 | Nutrients | Novel e-Health Applications for the Management of Cardiometabolic Risk Factors in Children and Adolescents in Greece | DOI: 10.3390/nu12051380 | Not paediatric diabetes |
| Tschaikner M, 2020 | IEEE Transactions on Biomedical Engineering | Novel Single-Site Device for Conjoined Glucose Sensing and Insulin Infusion: Performance Evaluation in Diabetes Patients During Home-Use | DOI: 10.1109/tbme.2019.2925434 | Not paediatric diabetes |
| Tumini S, 2020 | Pediatric Reports | Effectiveness and safety of flexible therapeutic schemes including first- and secondgeneration basal insulins during a paediatric summer diabetes camp | DOI: 10.4081/pr.2020.8254 | Not machine learning |
| Vaghefi E, 2020 | Diabet Med | THEIA™ development, and testing of artificial intelligence-based primary triage of diabetic retinopathy screening images in New Zealand | DOI: 10.1111/dme.14386 | Not paediatric diabetes |
| Wang TH, 2020 | BMC Public Health | Health information needs regarding diabetes mellitus in China: an internet-based analysis | DOI: 10.1186/s12889-020-09132-3 | Not paediatric diabetes |
| Watanabe H, 2020 | J Diabetes Investig | Preoperative fundus examination in patients with diabetes scheduled for surgery | DOI: 10.1111/jdi.13482 | Not paediatric diabetes |
| Wells BJ, 2020 | Diabetes Care | Detection of Diabetes Status and Type in Youth Using Electronic Health Records: The SEARCH for Diabetes in Youth Study | DOI: 10.2337/dc20-0063 | Not machine learning |
| Wong JJ, 2020 | Diabetes Technol Ther | Parental Perspectives: Identifying Profiles of Parental Attitudes and Barriers Related to Diabetes Device Use | DOI: 10.1089/dia.2019.0492 | Not machine learning |
| Wong JJ, 2020 | J Pediatr Psychol | Do Youth Want Psychosocial Screenings in Diabetes Clinic? Profiles of Acceptability | DOI: 10.1093/jpepsy/jsaa112 | Not machine learning |
| Wu Y, 2020 | Neural Netw | NFN＋: A novel network followed network for retinal vessel segmentation | DOI: 10.1016/j.neunet.2020.02.018 | Not paediatric diabetes |
| Xie Y, 2020 | Lancet Digit Health | Artificial intelligence for teleophthalmology-based diabetic retinopathy screening in a national programme: an economic analysis modelling study | DOI: 10.1016/S2589-7500(20)30060-1 | Not paediatric diabetes |
| Yang T, 2020 | JMIR Med Inform | Ensemble Learning Models Based on Noninvasive Features for Type 2 Diabetes Screening: Model Development and Validation | DOI: 10.2196/15431 | Not paediatric diabetes |
| Zhang D, 2020 | J Diabetes | TXNIP hypomethylation and its interaction with obesity and hypertriglyceridemia increase type 2 diabetes mellitus risk: A nested case-control study | DOI: 10.1111/1753-0407.13021 | Not paediatric diabetes |
| Zhao CY, 2020 | J Infect Dis | Microbiome data enhances predictive models of lung function in people with cystic fibrosis | DOI: 10.1093/infdis/jiaa655 | Not paediatric diabetes |
| Zheng M, 2020 | Cogn Res Princ Implic | How causal information affects decisions | DOI: 10.1186/s41235-020-0206-z | Not paediatric diabetes |
| Zheng C, 2020 | Transl Vis Sci Technol | Assessment of Generative Adversarial Networks Model for Synthetic Optical Coherence Tomography Images of Retinal Disorders | DOI: 10.1167/tvst.9.2.29 | Not paediatric diabetes |
| Erdal B, 2021 | Diabetic Medicine | The comparison of two mealtime insulin dosing algorithms for high and low glycaemic index meals in adolescents with type 1 diabetes | DOI: 10.1111/dme.14444 | Not machine learning |
| Schillinger D, 2021 | Health Serv Res | Employing computational linguistics techniques to identify limited patient health literacy: Findings from the ECLIPPSE study | DOI: 10.1111/1475-6773.13560 | Not paediatric diabetes |
| Szadkowska A, 2021 | Pediatric Diabetes | Impact of factory-calibrated Freestyle Libre System with new glucose algorithm measurement accuracy and clinical performance in children with type 1 diabetes during summer camp | DOI: 10.1111/pedi.13135 | Not machine learning |
| Calhoun P, na | Biometrics | Repeated measures random forests (RMRF): Identifying factors associated with nocturnal hypoglycemia | DOI: 10.1111/biom.13284 | Not paediatric diabetes |
| Kumar DA, na | Journal of Ambient Intelligence and Humanized Computing | Application of back propagation artificial neural network in detection and analysis of diabetes mellitus | DOI: 10.1007/s12652-020-02371-7 | Not paediatric diabetes |
