# Supplementary material for: The Reporting Quality of Machine Learning Studies on Pediatric Diabetes Mellitus: Systematic Review
Source: J Med Internet Res. 2024 Jan 19;26:e47430. doi: 10.2196/47430 (PMC10837761; doi:10.2196/47430)
Supplement: Multimedia Appendix 5 [file jmir_v26i1e47430_app5.docx]

**Table S5 Initial agreement of reviewer pairs during the screening of records and selection of full-text reports**

|  | **Screening of records** | | **Selection of full-text papers** | |
| --- | --- | --- | --- | --- |
| Revier pairs | Agreement | Kappa | Agreement | Kappa |
| 1 | 83% | 0.48 | 60% | 0.43 |
| 2 | 75% | 0.17 | 20% | 0.17 |
| 3 | 75% | 0.24 | 73% | 0.29 |
| 4 | 80% | 0.45 | 80% | 0.48 |
| 5 | 81% | 0.15 | 20% | 0.11 |
| 6 | 78% | 0.23 | 40% | 0.06 |
| 7 | 72% | 0.42 | 80% | 0.52 |
| 8 | 75% | 0.42 | 85% | 0.60 |
| 9 | 84% | 0.62 | 74% | 0.28 |
| 10 | 63% | 0.27 | 63% | 0.31 |
| 11 | 80% | 0.51 | 69% | 0.06 |
| 12 | 94% | 0.83 | 84% | 0.38 |
| Total | 80% | 0.47 | 67% | 0.30 |
