# Supplementary material for: The Reporting Quality of Machine Learning Studies on Pediatric Diabetes Mellitus: Systematic Review
Source: J Med Internet Res. 2024 Jan 19;26:e47430. doi: 10.2196/47430 (PMC10837761; doi:10.2196/47430)
Supplement: Multimedia Appendix 6 [file jmir_v26i1e47430_app6.docx]

**Table S6 Characteristics of studies assessed via MI-CLAIM (N=21)**

| **Variable** | **Category [references]** | **N** | **%** |
| --- | --- | --- | --- |
| Publication year | 2016 [66, 67, 68, 69] | 4 | 19.0 |
|  | 2017 [70, 21] | 2 | 9.5 |
|  | 2018 [71] | 1 | 4.8 |
|  | 2019 [72, 73, 74, 75, 76, 77] | 6 | 28.6 |
|  | 2020 [78, 79, 80, 81, 82, 83, 84] | 7 | 33.3 |
|  | 2021 [85] | 1 | 4.8 |
| Scientific field of the journal | Medical [21, 66, 68, 71, 74, 75, 78-81, 84, 85] | 12 | 57.1 |
|  | Engineering [67, 69, 70, 72, 73, 76, 77, 82, 83] | 9 | 42.9 |
| Study aim | Non-invasive hypolycaemia monitoring [21,67, 69, 70, 76] | 5 | 23.8 |
|  | Insulin bolus calculators for closed-loop glucose control [66,82-84] | 4 | 19.0 |
|  | Accurate glucose prediction from CGM data [72, 73, 78, 79] | 4 | 19.0 |
|  | Etiological or prognostic biomarkers of T1DM [77, 80, 81, 85] | 4 | 19.0 |
|  | Etiological or risk factors for insulin resistance or T2DM [71, 74, 75] | 3 | 14.3 |
|  | Other [68] | 1 | 4.8 |
| Involved subjects | Human [21, 67-71, 74-77, 79-81, 85] | 14 | 66.7 |
|  | Human and in silico [78] | 1 | 4.8 |
|  | In silico [66, 72, 73, 82-84] | 6 | 28.6 |
| Data type | Time series [66, 69, 70, 72, 73, 78, 79, 82-84] | 11 | 52.4 |
|  | Omics [21, 74, 77, 80, 80, 81, 85] | 6 | 28.6 |
|  | Other [68, 71, 75, 76] | 4 | 19.0 |
| Training sample size | ≤10 [67, 69,70,72,73,76,82-84] | 9 | 42.9 |
|  | 11-100 [21, 66, 75, 78, 79, 80] | 6 | 28.6 |
|  | >100 [68, 71, 74, 77, 81, 85] | 6 | 28.6 |
| Test sample size | ≤10 [67, 69, 70, 72, 73, 75, 76, 82-84] | 10 | 47.6 |
|  | 11-100 [21, 66, 71, 79, 80, 81, 85] | 7 | 33.3 |
|  | >100 [68, 74, 77, 78] | 4 | 19.0 |
| Separation of training and test data | Same patients - time series data [66m 72, 73, 76, 78, 82-84] | 8 | 38.1 |
|  | Same patients - cross-validation [21, 68, 77, 80] | 4 | 19.0 |
|  | Different patients - from the same centre [67, 69-71, 79] | 5 | 23.8 |
|  | Different patients - from muliple centres [74, 85] | 2 | 9.5 |
|  | Different patients - from external centres [75, 81] | 2 | 9.5 |
