# Supplementary material for: The Reporting Quality of Machine Learning Studies on Pediatric Diabetes Mellitus: Systematic Review
Source: J Med Internet Res. 2024 Jan 19;26:e47430. doi: 10.2196/47430 (PMC10837761; doi:10.2196/47430)
Supplement: Multimedia Appendix 7 [file jmir_v26i1e47430_app7.docx]

**Table S7 Assessment details of studies**

|  |  |  |  | **MI-CLAIM Items*** | | | | | | | | | | | | | | | | | | |  |  |
| --- | --- | --- | --- | --- | --- | --- | --- | --- | --- | --- | --- | --- | --- | --- | --- | --- | --- | --- | --- | --- | --- | --- | --- | --- |
| **Author, Year** | **Goal** | **Data** | **Subj** | **1.1** | **1.2** | **1.3** | **1.4** | **1.5** | **2.1** | **2.2** | **2.3** | **2.4** | **2.5** | **4.1** | **4.2** | **4.3** | **5.1** | **5.2** | **5.3** | **5.4** | **5.5** | **6.1** | **OC** | **OR** |
| Daskalaki E, 2016 [66] | BC | TS | MED | Y | Y | N | U | N | U | Y | N | U | US | U | U | N | Y | U | Y | Y | Y | T4 | D | A |
| Ling SH, 2016 [67] | HPN | TS | ENG | Y | Y | N | U | U | U | Y | Y | U | S | U | U | U | N | N | N | N | N | T4 | D | NN |
| Miller RG, 2016 [68] | O | O | MED | Y | Y | Y | Y | N | Y | Y | N | N | S | N | N | N | N | N | N | N | N | T4 | A | SA |
| Phyo Phyo San, 2016 [69] | HPN | TS | ENG | Y | Y | N | N | U | N | N | N | N | US | Y | Y | N | N | N | N | N | N | T4 | D | NN |
| Ling SH, 2017 [70] | HPN | TS | ENG | Y | Y | N | N | Y | N | N | Y | N | US | Y | N | N | N | N | N | N | N | T4 | D | A |
| Siegel AP, 2017 [21] | HPN | OM | MED | Y | Y | N | N | N | Y | N | N | Y | S | Y | U | U | U | N | U | N | U | T4 | NN | A |
| Stawiski K, 2018 [71] | T2R | O | MED | Y | Y | Y | Y | N | Y | Y | Y | Y | S | Y | U | U | Y | N | Y | U | U | T3 | SA | NN |
| De Bois M, 2019a [72] | CGM | TS | ENG | Y | Y | Y | U | N | Y | Y | U | Y | S | U | U | N | U | N | U | U | U | T4 | D | A |
| De Bois M, 2019b [73] | CGM | TS | ENG | Y | Y | Y | N | U | Y | Y | U | Y | S | N | U | N | U | N | U | N | N | T4 | D | A |
| Khusial RD, 2019 [74] | T2R | OM | MED | Y | Y | Y | U | U | Y | Y | Y | U | S | U | U | N | Y | N | Y | N | Y | T4 | NN | A |
| Langner T, 2019 [75] | T2R | O | MED | Y | Y | U | N | N | Y | Y | Y | U | US | U | U | N | Y | Y | Y | Y | Y | T1 | A | A |
| Ngo CQ, 2019 [76] | HPN | O | ENG | Y | Y | N | N | N | Y | N | N | Y | S | U | Y | N | N | N | N | N | N | T4 | D | NN |
| Stanfill B, 2019 [77] | T1P | OM | ENG | Y | Y | N | N | Y | Y | Y | N | U | S | Y | U | Y | Y | Y | U | N | N | T4 | NN | A |
| Amar Y, 2020 [78] | CGM | TS | MED | Y | Y | Y | Y | U | N | Y | N | Y | US | Y | Y | N | Y | N | Y | N | Y | T4 | A | A |
| Dave D, 2020 [79] | CGM | TS | MED | Y | U | N | N | N | Y | Y | N | Y | S | U | U | N | Y | N | Y | N | Y | T4 | A | A |
| Frohnert BI, 2020 [80] | T1P | OM | MED | Y | Y | N | U | Y | Y | U | N | Y | S | Y | Y | Y | Y | Y | Y | U | U | T4 | NN | D |
| Garavelli S, 2020 [81] | T1P | OM | MED | Y | Y | Y | Y | N | U | Y | Y | U | US | U | N | N | N | N | N | N | N | T4 | A | NN |
| Li K, 2020 [82] | BC | TS | ENG | Y | Y | N | U | N | Y | Y | U | N | US | N | N | Y | N | N | N | N | N | T4 | A | A |
| Zhu T, 2020 [83] | BC | TS | MED | Y | Y | U | U | Y | Y | U | U | Y | S | U | U | Y | U | N | N | N | Y | T4 | D | A |
| Zhu T, 2020 [84] | BC | TS | ENG | Y | Y | U | U | Y | Y | Y | U | Y | S | U | U | Y | U | N | N | N | N | T4 | D | A |
| Webb-Robertson BM, 2021 [85] | T1P | OM | MED | Y | Y | Y | U | N | Y | Y | Y | Y | S | Y | U | N | Y | Y | Y | U | U | U | SD | NN |

*MI-CLAIM items 1.1-6.1 –(see Appendix Table S4).

**A**: Agree**; BC**: insulin bolus calculator for closed-loop glucose control; **CGM**: accurate glucose level of hypoglycemia prediction from continuous glucose monitor data; **D**: Disagree; **ENG**: engineering; **HPN**: hypoglycemia prediction using non-invasive methods; **MED**: medical; **N**: No; **NN**: Neither agree, nor disagree; **O**: Other; **OC**: Overall expert assessment of clinical impact; **OM**: omics; **OR**: Overall expert assessment of replicability; **S**: structured data; **SA**: Strongly agree; **SD**: Strongly disagree; **Subj:**  Scimago subject category; **T1-T4**: Tier 1 – Tier 4; **T1P**: etiological or prognostic biomarkers for T1DM; **T2R**: etiological or risk factors for insulin resistance or T2DM; **TS**: glucose time series; **U**: unsure; **US**: unstructured data; **Y**: Yes
