# Supplementary material for: The Reporting Quality of Machine Learning Studies on Pediatric Diabetes Mellitus: Systematic Review
Source: J Med Internet Res. 2024 Jan 19;26:e47430. doi: 10.2196/47430 (PMC10837761; doi:10.2196/47430)
Supplement: Multimedia Appendix 8 [file jmir_v26i1e47430_app8.docx]

**Table S8. Summary of reported items in MI-CLAIM Part 1 (Study design)**

|  | **Study design (Part 1)** | | | | |
| --- | --- | --- | --- | --- | --- |
|  | **1.1** | **1.2** | **1.3** | **1.4** | **1.5** |
| Daskalaki E, 2016 [66] | Artificial pancreas system for T1DM | Develop a RL algorithm for personalized insulin dosing | - | - | - |
| Ling SH, 2016 [67] | Non-invasive HG monitoring in T1DM | Detect nocturnal HG from ECG signal via ELM-NN | - | - | - |
| Miller RG, 2016 [68] | CVD risk stratification of T1DM | Determine meaningful CVD risk groups via TSSA in T1DM | Baseline for follow-up: N, sex, race, Dg, center, Ref | All new / recent onset (<1 year) T1DM pts year seen in a single center between 1950-1980, followed up from 1986-88 for 25 years | - |
| Phyo Phyo San, 2016 [69] | Non-invasive HG monitoring in T1DM | Detect nocturnal HG from ECG signal via DBN | - | - | - |
| Ling SH, 2017 [70] | Non-invasive HG monitoring in T1DM | Detect nocturnal HG from ECG signal via MR-NLN with HPSOWM | - | - | Nocturnal CGM |
| Siegel AP, 2017 [21] | Non-invasive HG monitoring in T1DM | Detect HG from breath volatile organic compounds | - | - | - |
| Stawiski K, 2018 [71] | Measure insulin sensitivity in T1DM | Accurate insulin resistance estimator in T1DM | N, age-MD, sex, Dg, Ddur, Tanner stage, 12 clinical and metabolic variables | 50% T1DM pts (onset >1 year) from a single center, training / test cohort similarity shown with statistical tests | - |
| De Bois M, 2019a [72] | Accurate glucose prediction from CGM data | Compare the performance of 6 models in 30 min glucose prediction | Simulator software, N, Dg, parameters for meal frequency, CHO intake, insulin bolus, basal rate | - | - |
| De Bois M, 2019b [73] | Accurate glucose prediction from CGM data | Develop a new DCP model for 120 min glucose prediction | Simulator software, N, Dg, parameters for meal frequency, CHO intake, insulin bolus, basal rate | - | - |
| Khusial RD, 2019 [74] | NAFLD screening in children | Develop a NAFLD screening panel from blood and clinical data | N, age-MD, sex, Dg, race, Ref, 17 clinical and metabolic variables | - | - |
| Langner T, 2019 [75] | Quantify visceral adiposity from MRI scan | Develop a new method for the segmentation of visceral and subcutaneous fat | - | - | - |
| Ngo CQ, 2019 [76] | Non-invasive HG monitoring in T1DM | Detect nocturnal HG from EEG signal | - | - | - |
| Stanfill B, 2019 [77] | Classification algorithm for matched case-control studies | Develop a general data pre-processing algorithm to enable any classification method for MCC design | - | - | Compare six standard classification algorithms with / without the proposed pre-processing step |
| Amar Y, 2020 [78] | Accurate glucose prediction from CGM data | Develop a new GCN model for 30-60 min glucose prediction | A) N, age-MD, sex, Dg, Ddur, comorbidity, device, 3 metabolic variables  B) Simulation software, N, Dg, age group, meal frequency parameters | All pts with CGM & insulin pump treated in a single center in 3 years | - |
| Dave D, 2020 [79] | HG prediction from CGM data | _ | - | - | - |
| Frohnert BI, 2020 [80] | Biomarkers for IA and T1DM progression | Integrate genetic, immunologic, metabolomic, and proteomic biomarkers for hypothesis generation via ROFI-P3. | - | - | Compare ROFI-P3 to the standard and widely used RFE algorithm |
| Garavelli S, 2020 [81] | Biomarkers for T1DM progression | Find prognostic miRNA and immunometabolic biomarkers for T1DM | Train / Test1 / Test 2: N, age-MD, sex, Ddur, comorbidity, center, 6 metabolic variables | 150 T1DM pts & 47 healthy controls; Test 1 (N=18) & Test 2 (N=26) T1DM cohorts from separate centers, similarity between training / test cohorts demonstrated via statistical tests | - |
| Li K, 2020 [82] | Accurate glucose prediction from CGM data | Develop GluNet for 30-60 min glucose prediction | - | - | - |
| Zhu T, 2020 [83] | Artificial pancreas system for T1DM | Develop a DRL algorithm for mealtime insulin dosing | - | - | Compare DRL performance with published standard bolus calculator used in insulin pumps. |
| Zhu T, 2020 [84] | Artificial pancreas system for T1DM | Develop DRL algorithm for single hormone (insulin) and dual hormone (insulin + glucagon) glucose control | - | - | Compare DRL with standard LGS algorithm published and used in insulin pumps. |
| Webb-Robertson BM, 2021 [85] | Biomarkers for IA and T1DM progression | Integrative ML model to discover metabolomic clinical and genetic prognostic biomarkers for T1DM | N, age-MD, sex, Dg, FDR, center, Ref, IA status | - | - |

**MI-CLAIM items - 1.1** The clinical problem in which the model will be employed is clearly detailed in the paper; **1.2** The research question is clearly stated; **1.3** The characteristics of the cohorts (training and test sets) are detailed in the text; **1.4** The cohorts (training and test sets) are shown to be representative of real-world clinical settings; **1.5** The state-of-the-art solution used as a baseline for comparison has been identified and detailed.

**CHO:** carbohydrate; **DBN**: deep belief neural network; **DCP**: derivatives combination predictor; **Ddur**: disease duration; **Dg**: diagnosis; **DRL**: deep reinforcement learning; **ECG**: electrocardiogram; **EEG:** electroencephalogram; **ELM-NN:** extreme learning machine based feed-forward neural network; **FDR:** first degree relatives with T1DM; **GCN:** gradually connected neural network; **HG:** hypoglycemia; **HPSOWM**: hybrid particle swarm optimization with wavelet mutation; **IQR**: interquartile range; **IA**: islet autoimmunity; **IS**: insulin sensitivity; **LGS**: low glucose insulin suspension; **MCC**: matched case-control; **miRNA**: micro-ribonucleic acid; **MR-NLN**: multiple regression-based neural logic network; **MD**: mean and dispersion; **ML:** machine learning; **N:** sample size; **NAFLD**: non-alcoholic fatty liver disease; **R**: range; **Ref**: reference study / publication; **RL**: reinforcement learning; **RFE**: recursive feature elimination; **ROFI-P3**: repeated optimization for feature interpretation followed by posterior probability product ML approach; **T1DM**: type 1 diabetes mellitus; **TSSA**: tree structured survival analysis
