# Supplementary material for: The Reporting Quality of Machine Learning Studies on Pediatric Diabetes Mellitus: Systematic Review
Source: J Med Internet Res. 2024 Jan 19;26:e47430. doi: 10.2196/47430 (PMC10837761; doi:10.2196/47430)
Supplement: Multimedia Appendix 9 [file jmir_v26i1e47430_app9.docx]

**Table S9. Summary of reported items in MI-CLAIM Parts 2 & 3 (Data and optimization)**

|  | **Data and optimization (Parts 2 & 3)** | | | |
| --- | --- | --- | --- | --- |
|  | **2.1** | **2.2** | **2.3** | **2.4** |
| Daskalaki E, 2016 [66] | - | Transformation of input data to a normalized system state vector | - | - |
| Ling SH, 2016 [67] | - | Normalization of ECG parameters by patient | Random allocation of patients to training / test cohorts from the same center | - |
| Miller RG, 2016 [68] | Ref, center, and time of data collection; laboratory measurement methods, and metrics for 24 demographic, clinical and metabolic predictors | No transformations were applied | - | - |
| Phyo Phyo San, 2016 [69] | - | - | - | - |
| Ling SH, 2017 [70] | - | - | Random allocation of patients to training / validation / test cohorts from the same center | - |
| Siegel AP, 2017 [21] | Centers of data collection; breath sample collection, preservation, and GC / MS analysis methods; platform and data file format for omics data | - | - | Feature selection process via LDA^1^ for the final HG prediction model |
| Stawiski K, 2018 [71] | Centre of data collection, Ref for glucose disposal rate measurement method, metrics for 16 clinical and metabolic predictors | The dependent variable (GDR) was log-transformed | Random allocation of patients to training / test cohorts from the same center, stratified by dependent variable | Two NNs (different feature sets):1000 random models (link functions, layer structures); MARSpline (estimation parameters), reference model. Model evaluation: R^2^ and Bland-Altman plots. |
| De Bois M, 2019a [72] | Simulation parameters, length, frequency, and variables in time series data | Standardization of input data, rearrangement of time-series for prediction time horizon | - | Six models (FFNN, LSTM, ELM, SVR, GP-RBF, and GP-DP), model structure, activation function, optimization algorithm, learning and regularization hyperparameters. Model training, hyperparameter tuning and testing via CV on 3-way split data. |
| De Bois M, 2019b [73] | Simulation parameters, length, frequency, and variables in time series data | Standardization of input data, rearrangement of time-series for prediction time horizon | - | Seven models (FFNN, ELM, GP-DP, ACP, AWA, DCP1, DCP2), model structure, activation function, optimization algorithm, learning and regularization hyperparameters. Model training, hyperparameter tuning and testing via CV on 3-way split data. |
| Khusial RD, 2019 [74] | Ref, centers of data collection; NAFLD Dg methods and criteria; blood sample preservation and high-resolution metabolomics analysis methods; platform, data file format and annotation omics data; laboratory methods and metrics for clinical phenotype characterization | Methods for dimension reduction and feature selection from metabolomics data, missing data imputation | Random allocation of patients to training / validation / test cohorts from multiple centers | - |
| Langner T, 2019 [75] | Ref, centers of data collection, MRI scanner configuration and imaging protocol, image resolution, manual labelling procedure | Image pre-processing: size, background noise, contrast adjustment, intensity normalization, color channels | Random selection of test cohort from a different center | - |
| Ngo CQ, 2019 [76] | Centre of data collection; EEG device, electrode settings, sampling rate, unit of analysis and labelling rules; glucose measurement device | - | - | Bayesian neural network structure (input, hidden, and output node structure). Model selection via log evidence calculation (equation provided). |
| Stanfill B, 2019 [77] | Ref, center of data collection, number of variables and summary statistics of omics datasets | Pre-processing of matched pairs: centering each feature within pairs; log-transformation and feature selection from multiple omics datasets | - | - |
| Amar Y, 2020 [78] | - | Setting of prediction time-horizon, imputation for missing glucose values | - | 3 proposed GCN models (layer structure, parameters, loss functions), model optimization by trial and error; 4 comparator models (AR, FC, GBM, RF), package provided. |
| Dave D, 2020 [79] | CGM device, observation time and number of data points, glucose summary statistics; variables of insulin pump data | Description of 26 features extracted from CGM signal, feature selection | - | Two models explained: LR with LASSO, RF with VIP-based feature selection. Model tuning: optimal feature selection via CV: |
| Frohnert BI, 2020 [80] | Ref of data collection, Dg criteria, raw data for IA status over time, list of included SNPs in genomic analysis, platform, and center for metabolomic, proteomic, and immune marker analysis, list of clinical variables | - | - | ROFI-P3 (integration predictions from different datasets via different ML models): model and feature selection via CV. |
| Garavelli S, 2020 [81] | - | miRNA normalization and log transformation of change vs healthy cohort | Two test cohorts selected from two different centers | - |
| Li K, 2020 [82] | Ref for public human datasets / simulation parameters, length and frequency, variables, and their metrics in time series | Steps of data pre-processing listed: handling outliers, missing data imputation, computation of features, alignment for time series, label transform and recover method | - | - |
| Zhu T, 2020 [83] | Simulation parameters, length, sampling rate of time series. Variables are described as agent state vector. | - | - | Equations for DRL and standard bolus calculator. Process for two step model tuning (population, then personalized model) |
| Zhu T, 2020 [84] | Simulation parameters, length, sampling rate of time series. Variables are described as agent state vector. | Computations for the state vector parameters from input data | - | Equations for two DRL (single-hormone, dual-hormone) algorithms. Methods for ML model architecture selection. Algorithm for two step model tuning (population, then personalized model) |
| Webb-Robertson BM, 2021 [85] | Ref for data collection, centers and methods for IA measurement, definition of outcomes, list of clinical variables, genomics platform and center, metabolomics platform, center, and annotation | Missing data imputation, normalization, dimensionality reduction and feature selection for omics data | Random allocation of patients to training / test cohorts from multiple centers | ROFI-P3 (integration predictions from different datasets via different ML models): model and feature selection via CV. |

**MI-CLAIM items - 2.1** The origin of the data is described, and the original format is detailed in the paper; **2.2** Transformations of the data before it is applied to the proposed model are described; **2.3** The independence between training and test sets has been proven in the paper; **2.4** Details on the models that were evaluated, and the code developed to select the best model are provided.

**ACP:** artificial neural network combination predictor; **AWA:** adaptive weighted average fusion algorithm; **CGM:** continuous glucose monitor; **CV:** cross-validation; **DCP:** derivatives combination predictor; **Dg:** diagnosis / diagnostic; **DRL:** deep reinforcement learning; **ELM:** extreme learning machine; **FFNN:** feed-forward neural network; **GC/MS:** gas chromatography / mass spectrometry; **GDR:** glucose disposal rate; **GP-DP:** Gaussian process regression with dot-product kernel; **GP-RBF:** Gaussian process regression with radial basis function kernel; **HG:** hypoglycemia; **IA:** islet autoimmunity; **LASSO:** least absolute shrinkage and selection operator; **LDA^1^:** linear discriminant analysis; **LR:** logistic regression; **LSTM:** long short-term memory; **MARSplines:** multivariate adaptive regression splines; **miRNA:** micro ribonucleic acid; **ML:** machine learning; **NN:** neural network; **Ref:** reference study / publication; **RF:** random forest; **ROFI-P3:** repeated optimization for feature interpretation with posterior probability product; **SNP:** single nucleotide polymorphism; **SVR:** support vector regression; **VIP:** variable importance plot
