# Supplementary material for: The Reporting Quality of Machine Learning Studies on Pediatric Diabetes Mellitus: Systematic Review
Source: J Med Internet Res. 2024 Jan 19;26:e47430. doi: 10.2196/47430 (PMC10837761; doi:10.2196/47430)
Supplement: Multimedia Appendix 10 [file jmir_v26i1e47430_app10.docx]

**Table S10. Summary of reported items in MI-CLAIM Part 4 (Model performance)**

|  | **Model performance (Part 4)** |  |  |
| --- | --- | --- | --- |
|  | **4.1** | **4.2** | **4.3** |
| Daskalaki E, 2016 [66] | - | - | - |
| Ling SH, 2016 [67] | - | - | - |
| Miller RG, 2016 [68] | - | - | - |
| Phyo Phyo San, 2016 [69] | Sensitivity | Sensitivity | - |
| Ling SH, 2017 [70] | Gamma (0.6*sensitivity + 0.4*specificity) | - | - |
| Siegel AP, 2017 [21] | Accuracy (% correct classification) | - | - |
| Stawiski K, 2018 [71] | R^2^ | - | - |
| De Bois M, 2019a [72] | - | - | - |
| De Bois M, 2019b [73] | - | - | - |
| Khusial RD, 2019 [74] | - | - | - |
| Langner T, 2019 [75] | - | - | - |
| Ngo CQ, 2019 [76] | - | Sensitivity | - |
| Stanfill B, 2019 [77] | Accuracy (% correct classification) | - |  |
| Amar Y, 2020 [78] | RMSE | Percentage of predictions in C-E zones of the Clarke Error Grid (clinically hazard zones) | P<0.05, P<0.01, P<0.001 indicated for comparison metrics by pt subgroups vs baseline |
| Dave D, 2020 [79] | - | - | - |
| Frohnert BI, 2020 [80] | ROC AUC | Accuracy (% correct classification) | P-values for comparing ROC AUC curves |
| Garavelli S, 2020 [81] | - | - | - |
| Li K, 2020 [82] | - | - | P<0.05, P<0.01, and P<0.005 indicated for all performance metric comparisons vs proposed model |
| Zhu T, 2020 [83] | - | - | P<0.05 and P<0.01 indicated for all performance metric comparisons vs baseline |
| Zhu T, 2020 [84] | - | - | P<0.05 and P<0.01 indicated for all performance metric comparisons vs baseline |
| Webb-Robertson BM, 2021 [85] | ROC AUC | - | - |

**MI-CLAIM items -**  4.1 The primary metric selected to evaluate algorithm performance (e.g., AUC, F-score, etc.), including the justification for selection, has been clearly stated; 4.2 The primary metric selected to evaluate the clinical utility of the model (e.g., ppV, NNT, etc.), including the justification for selection, has been clearly stated; 4.3 The performance comparison between baseline and proposed model is presented with the appropriate statistical significance

**RMSE:** root mean square error; **ROC AUC:** receiver operating characteristics area under the curve.
