# Supplementary material for: The Reporting Quality of Machine Learning Studies on Pediatric Diabetes Mellitus: Systematic Review
Source: J Med Internet Res. 2024 Jan 19;26:e47430. doi: 10.2196/47430 (PMC10837761; doi:10.2196/47430)
Supplement: Multimedia Appendix 11 [file jmir_v26i1e47430_app11.docx]

**Table S11. Summary of reported items in MI-CLAIM Part 5 (Model examination)**

|  | **Model examination (Part 5)** | | | | |
| --- | --- | --- | --- | --- | --- |
|  | **5.1** | **5.2** | **5.3** | **5.4** | **5.5** |
| Daskalaki E, 2016 [66] | Model parameter changes over time were graphically explored for one subject | - | Model parameter changes over time and model performance were discussed | Model parameter changes over time were graphically explored for one subject | In silico patient models with / without SI variation were tested and discussed. |
| Ling SH, 2016 [67] | - | - | - | - | - |
| Miller RG, 2016 [68] | - | - | - | - | - |
| Phyo Phyo San, 2016 [69] | - | - | - | - | - |
| Ling SH, 2017 [70] | - | - | - | - | - |
| Siegel AP, 2017 [21] | - | - | - | - | - |
| Stawiski K, 2018 [71] | Model performance vs ground truth values at extreme values were explored via Bland-Altman plots. | - | Prediction error of proposed and reference model vs ground truth was discussed for extreme values and different age-groups | - | - |
| De Bois M, 2019a [72] | - | - | - | - | - |
| De Bois M, 2019b [73] | - | - | - | - | - |
| Khusial RD, 2019 [74] | The selected clinical and metabolomic markers with best classification performance were presented | - | Results of sensitivity analysis of classification performance using various marker sets was discussed. | - | Results of sensitivity analysis of classification performance using various marker sets was discussed. |
| Langner T, 2019 [75] | Prediction error plotted vs ground truth volume of SAT and VAT- | MRI images of cases with best, median, and worst prediction errors for VAT and SAT volume were presented | Potential reasons for poor performance and location of mislabeled voxels were discussed. | MRI images of cases with best, median, and worst prediction errors for VAT and SAT volume were presented | Results on the training and test cohorts with different patient demographics and MRI device settings were discussed. |
| Ngo CQ, 2019 [76] | - | - |  | - | - |
| Stanfill B, 2019 [77] | A list of most influential lipidomic, metabolomic and genomic markers were presented with their ranks for two classification methods | A list of most influential lipidomic, metabolomic and genomic markers were presented with their ranks for two classification methods |  | - | - |
| Amar Y, 2020 [78] | Model performance was evaluated in patient-subgroups by age and glucose control parameters | - | Model performance was discussed vs state-of the art model in high-risk patients (high prediction errors, high risk for HG, high glucose variability) | - | Model performance was evaluated in patient-subgroups by age and glucose control parameters |
| Dave D, 2020 [79] | Transparent models were used. Methods of feature selection: LASSO for LR, VIP for RF. Selected features via LASSO and selected features with VIP for RF were presented. | - | Different feature sets and predictive performance in daytime vs nocturnal HG were discussed. | - | Predictive performance in daytime vs nocturnal HG were discussed. |
| Frohnert BI, 2020 [80] | Selected proteomic, metabolomic, genomic and clinical markers with best classification performance are presented. | Levels of features discriminating the control group vs cases were compared in graphs. | Selected features and their pathophysiological role were extensively discussed. | - | - |
| Garavelli S, 2020 [81] | - | - | - | - | - |
| Li K, 2020 [82] | - | - | - | - | - |
| Zhu T, 2020 [83] | - | - | - | - | In silico patient models with increased parameter variability were tested and discussed. |
| Zhu T, 2020 [84] | - | - | - | - | - |
| Webb-Robertson BM, 2021 [85] | Feature importance plots for selected genetic, environmental and metabolomic markers are presented. | Levels of features discriminating the control group vs cases were compared in graphs. | The pathophysiological role of selected features and associated metabolic pathways was extensively discussed. | - | - |

**MI-CLAIM items - 5.1** Examination technique 1; **5.2** Examination technique 2; **5.3** A discussion of the relevance of the examination results with respect to model/algorithm performance is presented; **5.4** A discussion of the feasibility and significance of model interpretability at the case level if examination methods are uninterpretable is presented; **5.5** A discussion of the reliability and robustness of the model as the underlying data distribution shifts is included

**HG:** hypoglycemia; **IA:** islet autoimmunity; **LASSO:** least absolute shrinkage and selection operator; **LR:** logistic regression; **MRI:** magnetic resonance imaging; **RF:** random forest; **SAT:** subcutaneous adipose tissue; **SI:** insulin sensitivity; **VIP:** variable importance plot; **VAT:** visceral adipose tissue
