# Supplementary material for: The Reporting Quality of Machine Learning Studies on Pediatric Diabetes Mellitus: Systematic Review
Source: J Med Internet Res. 2024 Jan 19;26:e47430. doi: 10.2196/47430 (PMC10837761; doi:10.2196/47430)
Supplement: Multimedia Appendix 12 [file jmir_v26i1e47430_app12.docx]

**Table S12. Additional searches in PubMed (Date: February 17^th^, 2021)**

| **#** | **Topic** | **Search term** | **Hits** |
| --- | --- | --- | --- |
| #1 | Machine Learning | ("Machine learning"[Title/Abstract]) AND (("1900/01/01"[Date - Publication] : "2021/02/17"[Date - Publication])) | 42,840 |
| #2 | Machine Learning Study | ("Machine learning study"[Title/Abstract]) AND (("1900/01/01"[Date - Publication] : "2021/02/17"[Date - Publication])) | 101 |
